# Methodological weaknesses in the measurement approaches and concept of housing affordability used in housing research: A qualitative study

**DOI:** 10.1371/journal.pone.0221246

**Published:** 2019-08-30

**Authors:** Ikenna Stephen Ezennia, Sebnem Onal Hoskara

**Affiliations:** Department of Architecture, Faculty of Architecture, Eastern Mediterranean University, North Cyprus, Famagusta, Mersin, Turkey; Politecnico di Milano, ITALY

## Abstract

Housing affordability (HA) is considered an important determinant of a country’s socioeconomic development and stability. However, its optimal measurement has remained a major concern worldwide. In recent decades, methodological development and researches on housing affordability measurement approaches (HAMA) have accelerated and continue to grow exponentially. Despite this intensive global development, very few attempts have been made to present the theoretical bases and track the developmental trends of these approaches. Thus, this study attempts to fill this literature gap and extend prior studies by exploring several alternatives of HAMA; with a focus on their methodological weaknesses. This paper highlights three emerging novel methodologies, which complement the relative strength of the conventional approaches. Findings suggest that the main research evidence which summarizes the weaknesses in the conventional measures is; their inability to incorporate sustainability features and over reliance on economic dimension. While the complexity of emerging methodologies, though deepen the overall understanding of multiple concerns that breed HA problems. But are less transparent, more data intensive, and their computation is very demanding, with a high tendency that their complex nature could weaken their uptake by researchers. This study raises concern over the nearly sole reliance on the conventional approaches in the reformations of policy instrument, despite their overwhelming weaknesses. It reiterates the need for reconsideration and offers new insight, but not conclusive information on better ways to conceptualize and measure HA.

## Introduction

There is a growing scholarly concern for the development of better methodologies that could optimally measure housing affordability (HA), as part of efforts towards addressing the ever escalating housing cost. These concerns are targeted towards achieving a wider range of positive policy and economic outcomes, such as; enhanced housing and transportation infrastructure, income adequacy, household wellbeing, reduced inequalities and improved rental housing [1]. These discussions among others, are the major issues that continue to steer HA at the core of several international discourses on policies related to housing. However, as discussions over HA issues continue, there is no consensus on the concept based on its meaning and measurement approach. Several arguments hold that this could be the result of the multiple views employed in its analysis [2], which produces a different outcome depending on what constitutes the approach [3].

Therefore, if debates on HA must be sought to address rising costs escalations, then the conventional definitions and traditional methods of measuring HA must be re-examined. Undoubtedly, HA must be viewed holistically with respect to the general ecosystem and should be responsive to sustainability issues [4,5]. It is noteworthy to mention that a growing number of studies have advanced the conventional measures, and the scope transcends poverty measurement and mere economic considerations [6,7]. Academic interest in HAMA has increased extensively, particularly as new methods are developed and older ones improved. Several approaches have been developed in this field. Despite this intensive development worldwide, prior surveys were not able to keep pace and little efforts have been made, to present the theoretical basis and developmental trends of various HAMA. Therefore, it is the conviction of the authors that there is a need for a new qualitative inquiry to consolidate current studies undertaken in this field. In this regard, a review of 98 scholarly journal articles indexed in Web of Science Core Collection, Google and ProQuest was conducted. Consequently, this study documents the exponentially growing academic interest in HAMA, and reviews the various studies exploring the concept, debates and challenges in undertaking HA analysis.

### Aim and article structure

This study neither aims to construct a new approach, nor rectify the weaknesses in various approaches, but to track, through an extensive literature review, the extent to which housing researchers have advanced methodologically in innovating better alternative methods for improved measurement outcome. The objectives are to present descriptions of identified methods and the continuing discussions on their relative suitability as affordability measures. For instance, some researchers advocate for complete replacement of the normative measures and have either proposed or developed alternative methods [6,7,8] that account for their weaknesses [3]. Others have modified these normative measures [9,10]. While, some argue for their continuous usage due to ease of application, global acceptance and common sense appeal [11,12]. This article articulates these debates and narrates the situation with review on HAMA and their weaknesses based on the main research question: What are the methodological weaknesses in the various HAMA as identified by researchers over the last few decades? From this, the following four sub-questions emerge: (1) What are the conceptual irregularities arising from the widely accepted definitions of HA? (2) What are the recent methodological discourses on the current worsening affordability trends across the globe and policy responses to quell them? (3) Which methods could serve as an alternative to the conventional approaches? And (4) What are the various procedures for improving HAMA as proposed by housing researchers?

The answers to these sub-questions will present sound evidence on the developmental trends of diverse approaches, as well as their suitability as affordability standards. This will permit clearer theoretical explanations of the identified approaches. In the end, the study constitutes a firm background for methodological discussions and proffers insight into future directions in HA agenda. By harmonizing wide collection of research in this field over a relatively long period, this article makes a valuable contribution to HA literature. It demonstrates that HA concept has evolved from its original meaning, which was focused on economic terms; to broader dimensions incorporating social and environmental criteria. And that incorporating wide ranging criteria that impact on households quality of life leads to better measurement outcome. Most key authors have embraced this evolution and view the change as positive.

The rest of this study is structured accordingly; Second Section describes the review search methods. Third Section discusses the literature search results based on research questions. Forth Section discusses the literature review findings and presents suggestions for improving HAMA and directions for future research, based on research aim, purpose and question. Fifth Section presents conclusion based on the contribution of the study to the international HA literature.

## Review search methods

### Study selection procedure

A review of related literature was undertaken between December 2016 and June 2018. Assessment methods of HA were investigated leading to an understanding of the weaknesses, strengths, uses and limitations associated with each method. The reports of empirical studies from peer-reviewed academic journals were given priority. The main for inclusion criteria for article selection is that the content significantly discusses HA concept and/or measurement approach (see S1 Table in Supporting Information). In the event of uncertainty, the complete text of the paper was read by the researchers. In addition, review papers, book reviews, non-empirical articles, news items, non-English publication, duplicates, monographs, editorials and encyclopedia articles were excluded from the data collection technique. Furthermore, housing affordability indexes developed by professional bodies and housing counselors were not considered, since they are not readily used by housing researchers.

A wide publication era was considered to reflect significant sources and historical materials that are relevant in forming the objective of this study. The time frame of this study is within 2000 and 2018, which is synonymous with the development of measures for assessing housing needs, problems and the calculation of affordable housing areas; that are the hallmark of the Millennium Development Goals (MDGs) Target 11 of Goal 7; relevant to all 191 United Nations (UN) member states.

### Searching procedures

**Fig 1:**
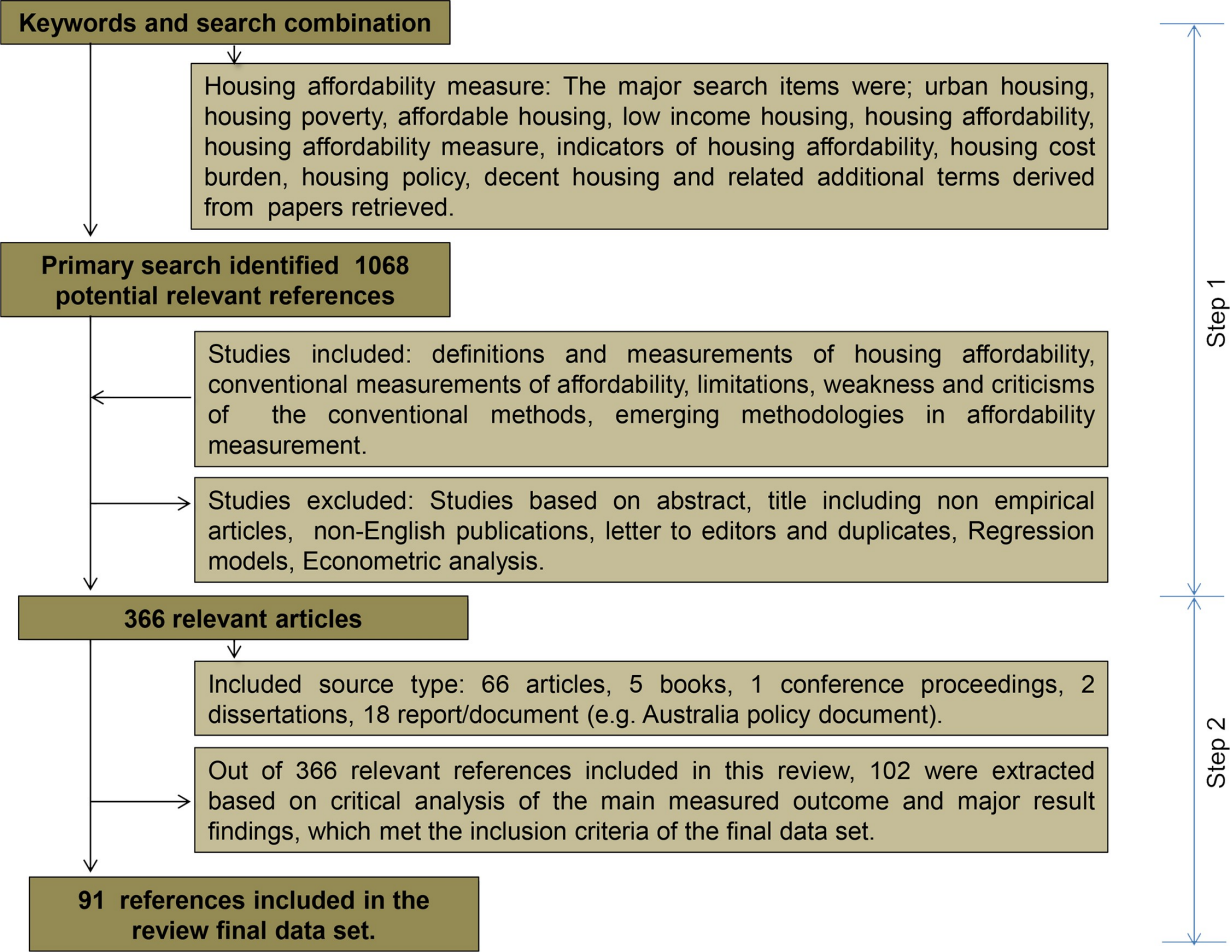
The literature review flowchart.

The literature search was grouped into two steps, as shown in Fig 1. Firstly, a wide-ranging search was conducted for common measurement approaches in title, abstract, and keywords used for related publications in HA research. In the second step, the ‘‘snowball” method of scanning through the reference list of relevant articles and the most recent published papers for related papers as well as previous studies they cited, was used to obtain relevant articles which significantly influenced the subject under review, since the search results of most databases were recently published and frequently cited articles. The main search terms include: “*affordable housing*, *housing affordability concept*, *housing affordability measure*, *indicators of housing affordability*, *measures of housing need”;* but were not entirely restricted to the aforementioned.

## Literature search results

As of June 2017, the initial search results yielded 1068 references. Further scrutiny of potentially relevant materials under investigation based on methodological decision analysis produced a total of 91 references. 65 were journal articles, 01 conference papers, 05 books, 03 dissertations, 18 reports/documents (e.g. Australian policy document). An extraction data sheet was prepared to illustrate the final data set. (see S2 Table in Supporting Information). These references were retrieved from ProQuest (n = 04); Web of Science (n = 61); and Google Scholar (n = 26) online search engines (see S2 Table in Supporting information).

### Meaning and definitions of housing affordability

The phrase “housing affordability” (HA) is polysemous in meaning, because it is used to describe several components of housing needs such as housing condition, housing costs, housing quality, household income and overcrowding. HA has become a multi-faceted phrase [13] due to its heuristic nature. It has been perceived differently by several researchers who have used various definitions and methodological approaches in measuring it [14]. However, HA is generally described as households’ ability to access and obtain decent housing without experiencing unwarranted financial hardship [15,16]. Such a broad description refers to two aspects: (1) Attainability—access to a house at a certain period and; (2) Sustainability—the possibility of the household to continue maintaining the house. This implies the ability (or inability) to sustain economic commitments with regard to the housing already obtained [2].

#### Conceptual irregularities in housing affordability definitions

Earlier attempts by researchers to define HA were characterized by diverse interpretations which focused primarily on the economic dimension; as illustrated in Table 1. For instance, Howenstine’s [17] interpretation of HA as an ‘unreasonable amount’ was faulted by Maclennan and William, [18] whose definition, though clarified the question of ‘unreasonable amount’ but their concept of a ‘given standard of housing’ and ‘unreasonable burden’ was also not comprehensive. Hancock [21] introduced the concept of opportunity cost. This implies trade-offs made by households in order to afford housing cost and whether such trade-offs is reasonable or excessive. The weakness in the concept of opportunity cost is that it created an understanding of HA that does not imply any form of measurement approach.

**Table 1:** Selected key definitions of housing affordability based on literature.

References	Focus	Definition
[17]	Economic	Households ability to acquire decent accommodation by the payment of a reasonable amount of its income on shelter
[18]	Economic	Affordability is about securing some prescribed housing standard (or different standards) at a cost (rent or price) which exerts no unreasonable burden on household incomes, according to any third party (mostly the government).
[19]	Economic	The ability households to occupy housing that meets socially acceptable standards of adequacy, considering household composition (size and type) at a net cost which allows them sufficient income for survival without plunging them below some poverty standard.
[20]	Economic	Focuses on the housing expenditure-household income relationship, and thus seek to design, a measure that can establish what amount of rent spent on the housing that is considered affordable.
[21]	Economic	Affordability is about the concept of opportunity cost of housing, what is forgone in order to secure housing and if that which is forgone is unreasonable or moderate in some sense.
[22]	Economic	Households are experiencing affordability burden, if the cost of housing displaces excessively other expenses.
[23]	Socio-economic	Affordability describes the ability of households to meet the costs of housing, while there is the possibility of maintaining other basic expenses.
[11]	Socio-economic	Housing affordability is the articulation of the challenges that confront households in balancing the actual or potential housing cost, as well as the non-housing expenses, within the limits of their income.
[24]	Socio-economic	Affordability is a broad concept that is concerned with housing appropriateness and standards, as well as social and neighborhood issues, in addition to economic participation.
[6,7]	Social, Economic & Environmental	Affordability is comprised of some broader and more sustainable perceptions of wide ranging criteria such as economic, environmental and social aspects that affect households.
[25]	Social, Economic & Environmental	The housing affordability concept should receive both social and economic content, in addition to the ecological content.

Only a few studies attempted to distinguish the HA concept from its measurement approach. A very clear example is the views of Chapman [26], who opined that HA is the measure of the financial outcome of outright purchase or renting a house. Recently, researchers began to see the need to consider other non-monetary dimensions into the definitions and measurement approaches of housing affordability. For instance, Leishman and Rowley [24] posited that HA is comprised of housing standards and appropriateness, as well as social, neighborhood issues and economic participation. However, Rowley and Ong [27] questioned the extent to which neighborhood quality is addressed when evaluating the appropriateness of affordable housing with regards to cost.

#### Housing affordability concept–Weaknesses and new understanding

Indeed, the debates, concerns and opinions about HA concept reflect the different assumptions and priorities of researchers with different orientation. For instance, economists mostly prioritize clarity of concept, utility, and objectivity [28], while sociologists usually focus on social inequality concerns and the research capacity of HA to cover actual experiences of household housing stress [29]. Architects are focused largely on providing savings and cost reductions in both upfront costs and the ongoing cost of occupation [30]. Such diverse academic orientation led to the revelation of the weaknesses in the conventional measurement approaches, and arguments in support of methodologies that better reflect the concept of HA.

However, there are no generally agreed standards by which it is conceived or measured. Thus, international housing policy documents of most countries adopt the 'rule of thumb', advocating that 30 percent or more of household income should be spent on housing for it to be considered affordable [31]. This notion is usually propagated without any recourse to household composition, size, housing quality or neighborhood characteristics, income levels, age groups and location. Therefore, the 30% affordability standard as a qualifying ratio is flawed. But it has remained the reference point for housing policy’s purposes; for instance, in allocating housing vouchers, low-income tax credits, stamp duty concessions and grants.

The most common weakness in the HA concept is its insensitivity to the effect of housing supply; and neglect for people’s heterogeneous behaviors within the same income group. This is consistent with Stone et al., [32] assertion that the HA concept must be founded on the interaction flanked by households and their houses. The authors maintained that affordability is not an innate attribute of the house and its measurement must not depend upon house price and income alone; owning that an affordable house could be different for each individual. Therefore, it suggests that in measuring affordability the approach applied must significantly capture each human variable that describes such relational concept.

#### Adopting sustainability principles into measurement criteria of HA

**Fig 2:**
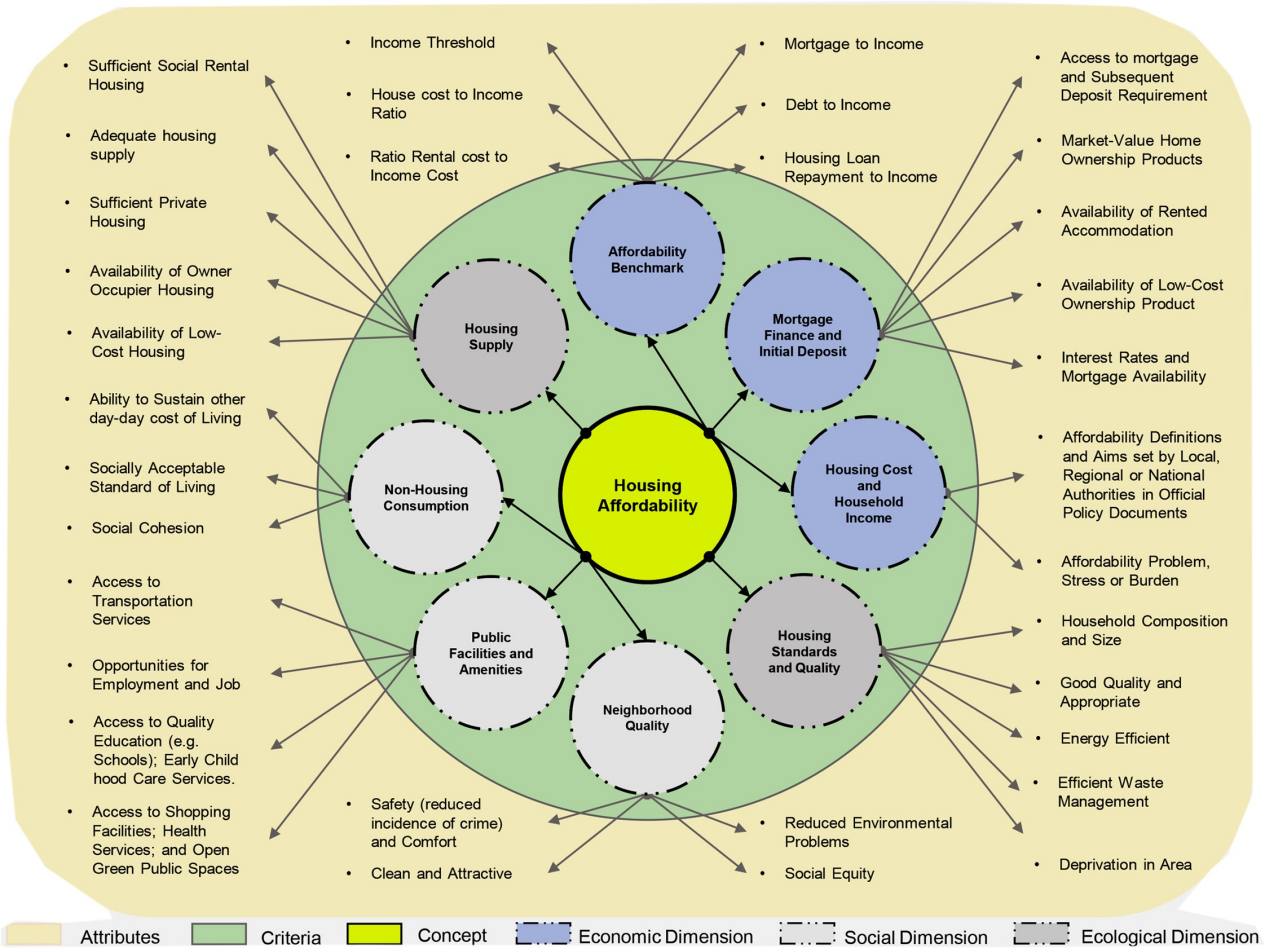
A conceptual model for understanding the HA concept. (Source: Adapted from [4,6,7].

Recent studies are starting to consider wider dimensions of the criteria that induce HA problems and have advocated that HA assessments should address more sustainable and wide ranging criteria such as economic, environmental and social aspects that affect households [6.7]. This implies that a broader range of quantitative and qualitative criteria must be accounted for, towards achieving actual HA. These include, but not limited to, social wellbeing, neighborhood and location issues. In addition to sustainability and health concerns, housing standards and appropriateness, housing market, transportation cost, households and their quality of life as well as political criteria [33,27,34] instead of exclusively focusing on income and housing price as the prime determinants. Congruently, it is essential that both sustainability and affordability issues are tackled simultaneously in the measurement approaches used in HA analysis [7]. These are illustrated in Fig 2, a model for understanding the evolving concept of HA.

### Current affordability trends and weaknesses in policy responses

Recent discourse on HA are focused on whether meager income and/or issues of housing inadequacy trigger HA problems. For instance, as shown in Table 2 Americans (US) public policy is guided on the perception that HA issues are problems of poverty [35,18], and inadequate housing triggering a 3% worse-case needs [36]. Hence, solutions have been addressed on the side of demand policies, such as eliminating regulatory barriers. However, merely removing regulatory barriers might be of limited benefit to low-income households, supposing that housing developers continually focus on luxury houses [37]. In nearly all countries of Europe and the United Kingdom, policy framings are based on concerns that HA problems are caused by insufficiency of affordable housing supply; and that the lower income households suffer affordability stress the most, even though they pay for cheaper houses [38]. Therefore, have sought solutions on the supply side with increased housing provision, and development of urban planning to ensure that housing supply respond better to changing demands [39]. However, the over dependence on the private sector to supply more housing units weakened the potency of the policy changes; coupled with the adoption of America’s (US) “demand-side” policy which did not exert meaningful impact to reverse the UK’s worsening volatile housing prices, nor improve overall affordability, that is occasioned by demand pressure through in-migration [39].

**Table 2:** Summary of key policy responses of selected countries and their attempts in addressing worsening housing affordability problem.

Perceived key triggers of housing affordability problem	Orientation of affordability Policy (Solutions sought)	Key Housing policy initiative	Enactment Date	Country	Major weaknesses in the policy strategies
Poverty. Limited housing supply. Lack of decent quality affordable housing. Lack of sufficient rental housing for low-income populace. Housing market volatility and changes in income distribution. Changing regulatory regime that impedes large-scale development in expensive locations. Not as a result of declining availability of land.	Solutions sought through the side of demand policies. Removing regulatory barriers. Encouraging infrastructure investment. Self-Help approaches. Encouraging access to affordable housing, for both owners and renters. Restructuring assistance for the homeless. Changes in the regulations of housing supply.	Low Income Housing Tax Credit (LIHTC) HOPE VI Program Housing Choice Voucher (HCV) program Hardest Hit Program	1987 1993 1998 2010	USA	Removing regulatory barriers alone might be of limited benefit, if the focus of housing developers is fixated on developing luxury houses. Current federal expenditure on housing is weakly targeted, because of huge amounts it spends on the mortgage interest deduction, which essentially profits the wealthy homeowners. Current political will in allocating significant new resources to check this dilemma is extremely weak. Rents in housing financed with tax credits are fixated to a given sum, so the percentage of income paid on housing by tenants may increase if their incomes decline and could spend over 30 percent of their income on rent. The normative policy agenda ought to focus on better understanding of the benefits of limits and costs of new construction.
Insufficiency in affordable housing supply. Demand pressure caused by in-migration. Affordability brunt is borne more by the low-income households. Failures of housing market occasioned by pro-market reforms.	Solutions sought through the side of supply policies. Increasing new housing supply. Urban planning development Making supply of housing more sensitive to market conditions.	Housing Green Paper Sustainable Communities: Building for the Future Starter Home Initiative	2000 2003 2015	UK	Marred by the over dependence on the private sector for the provision of additional housing units. Wrong adoption of America’s “demand-side” policy. Higher income tenants occupy some of the cheaper housing targeted by tax credits, and this weakens the policy rationale for such supply-side measures. New build cannot totally address housing affordability, also managing and modernizing existing housing stock must be considered. Starter home is a short-term initiative that fails to address the main issues behind affordability problem. Merely increasing housing supply alone creates its own demand.
Not necessarily a problem of demand and supply. Rather fiscal and financial problems. Housing system Imperfections Growing inequality in income and wealth	Geared towards restrictions of housing purchase. Suppression of housing markets speculation. Adopting strict administrative measures. Creating market-oriented housing system. Stimulating affordable housing investment. Promoting housing subsidies.	Capped-Price Housing (CPH) also known as dual-restriction commodity. Public Rental Housing (PRH) Home Purchase Restriction (HPR) Policy.	2007 2007 state level and in 2010 nation-wide 2010–2015 Re-enacted back in 2016	China	Focused essentially on sale volume. Restrictions on housing purchases can only suppress housing prices in the short period. Perceived as an ineffective approach in controlling rising housing prices. Only postpones certain categories of demand for the future instead of total elimination. Beclouded with uncertainty in terms of its suitability. Not founded on the reasons behind high housing prices. Not concerned on the impact it exerts on housing markets. Raises entry cost into housing market. Price of housing is not determined by the effective households’ demands.
Lack of funds. Poor access to finance. Rising poverty due to migration occasioned by political instability in the Middle East. Housing system Imperfections Growing inequality in income and wealth	Solutions sought through policy reforms to encourage access to homeownership. Liberalization and deregulation of institutional and legal framework as it concerns the control of urban development. Tax exemption and reduced vat rate. Discouraging luxury housing through additional tax. Financial subsidy and incentive.	Mass Housing Fund Planned Urbanization and Housing Production Program Tenth National Development Plan KENTGES Integrated Urban Development Strategy and Action Plan	1980–1998 2002–2014 2014–2018 2010–2023	Turkey	Budget deficit and inefficiency in the mortgage markets weakened the potency this policy. Encouraged the growth of shanty and luxury homes rather than the much needed social and mass housing. Unable to develop institutional form that could deliver housing to the targeted populace. Policies are formulated non-theoretically.
Lack of funds. Poor access to finance and insufficient financial mechanism. Slow administrative procedures and cumbersome regulatory approval process. Increase in speculation and inflation. Dearth of housing integrated planning and programs. Very small amount of mortgage lending institutions.	Fiscal policies border on increasing governmental spending. Solutions sought through policy reforms to encourage access to homeownership. Provision of tax holiday for housing developers To reduce the number of individuals excluded from financial services. Addressing legislative bottlenecks shredding housing. Delivering at least one million decent affordable housing units annually. Establishing a new mortgage regime and developing secondary mortgage market. Negotiating more favorable mortgage terms.	National Policy on Housing (NHP). National Transformation Agenda. Final Draft Nigeria Land, Housing and Urban Development Roadmap.	2006 2011–2015 2014–2043	Nigeria	Increased governmental spending alone cannot guarantee affordability as various players in the financial sector could hit back through trade and exchange rate fluctuations, leading to continuous weakening of the currency’s purchasing power. The federal and state government is expected to carter for about 50% of the housing supply deficit; while the private sector covers the rest. Current trend of leaving the provision of housing to the dictates of market forces cannot support affordable housing. Housing prices could be increased by easy credit policy.

In China, the major causes of high housing prices may not necessarily be the problem of demand and supply, rather fiscal and financial problems. Hence, policy framings are geared towards the restriction of housing purchase [40]. However, uncertainty still blights the appropriateness of such policy on the Chinese housing market since its implementation, with some researchers arguing against it, noting that it was not founded on the main reasons for high housing prices, and shrinks trade volume in housing market [41]; while others argue in its favor, noting also that it decreased the growing levels of housing prices [40]. Similarly, in Turkey and almost all developing countries, policy responses are predicated upon the fact that HA dilemmas are issues of inadequate funding and access to finance. Solutions have been sought on reforming policy frameworks to encourage access to homeownership, taking into account that running cost affordability may be problematic for many low-income homeowners [42,43]. In the Nigerian context, fiscal policies border on increasing governmental spending only [44]. This implies that more money is available to households and therefore, households have a leveraged purchasing power. More income for households would mean that, affordability would also rise proportionately. However, the weakness in this policy response is that it does not guarantee affordability as various stakeholders in the financial sector could hit back through trade and exchange rate fluctuations, thus resulting in the continued weakening of the Nigerian currency’s purchasing power, as with the Turkish currency.

The worsening HA problems experienced across the globe seem to defile solutions. Many of the policy responses discussed above failed to address the issues for which they were enacted. This could be attributed partially to hastily affordability analysis founded on the conventional measurement approaches. An ill-defined and poorly measured affordability dilemma will always lead to inappropriate policy response that may have little or no effect in ameliorating affordability stress suffered by residents. Therefore, considering several dimensions (rather than just economic) in the measurement approaches of the criteria that influence affordability burden could perhaps be a major step in tackling this ever growing monster.

### Housing affordability measurement approaches (HAMA)

**Fig 3:**
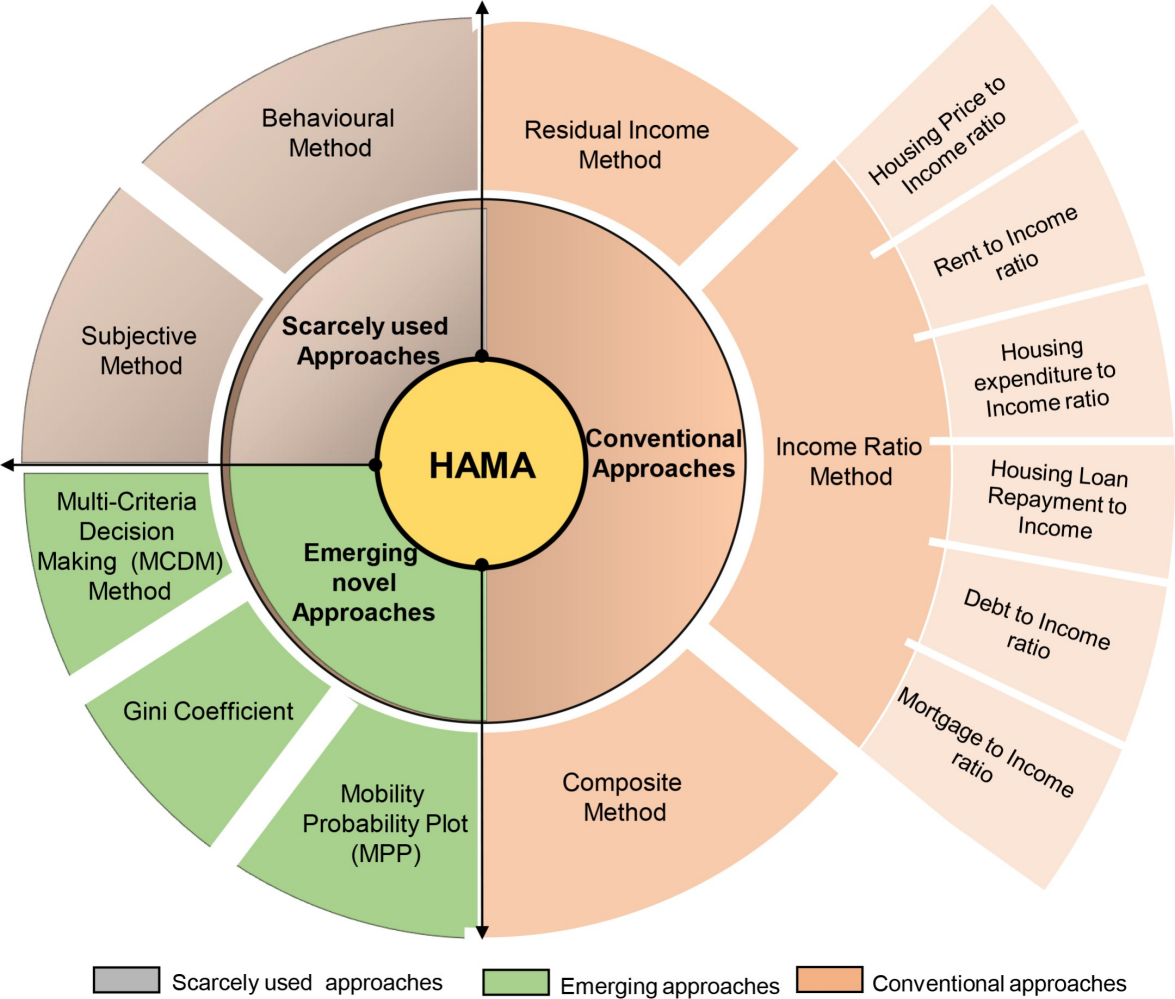
Classification of HAMA based on literature survey.

HAMA that is employed by housing researchers can be broadly classified into three distinctive approaches. According to their frequency of application and developmental trend such as conventional approach, scarcely used approach, and emerging innovative approach. This is shown in Fig 3. Each approach is unique, but fundamentally describes the assumptions of a reasonable payment for housing [45] and the interaction between income and housing cost [3]; as well as the ability for mortgage repayment [46]. However, measuring HA based on the capability to meet loan/mortgage requirements is generally flawed, due to the leniency of qualification criteria for a mortgage [47]. Researchers have recently argued that HA can only be measured in comparison to income. Yet, many professionals, journalists and analysts often use rents or housing prices and/or the increments of both with no references to income in describing HA. The weakness in such description is that prices alone and trends in house prices or rent posses only anecdotal value, but are no indicator of HA without any form of comparison to incomes [38]. This is because when income rises proportionately with house price, HA remains unchanged. There are several other technical and methodological weaknesses regarding HAMA as illustrated in Table 3. These weaknesses largely reflect what constitutes costs of housing and household income measurement [3,45] collective concern about survey data quality [48] profound insensitivity to other costs linked with housing quality and choice [33,14].

**Table 3:** Summary of findings based on the literature reviewed.

Approach/Method		Strengths	Weaknesses	Key Variables Used	Common Application Areas
Conventional (Normative) Approaches	IRM	Ease of application. Ease of affordability comparisons across regions. Based on a small number of regularly available variables. Enjoys global acceptance. Applicable in a range of areas to study the differences across households and affordability trends over time. Measures the actual household expenditure relative to their actual income.	Arbitrary benchmark, no clear rationale behind affordability thresholds. Does not account for household structure and level of income of various families, unless modified. Does not address housing quality and adequacy. Concentrates on economic aspects only. Erroneously assumes that every family and individual has equal capacity to pay for housing. Fails to address non-housing expenditure. Could over and under estimate affordability problems.	House price. Monthly gross income. Monthly rent.	Ascertaining the market realities in housing and income. Comparison of same type of household affordability over different areas in a given time frame. Describing household expenditure. Eligibility criteria for public housing subsidy allocation. Predicting household ability for housing payment.
	RIM	Effective where the economic realities are similar for all chosen samples. Considers household structure and different levels of income among different households. Sensitive to housing market realities in income and housing. Acknowledges that people have different spending needs. Addresses equity concerns. Incorporates housing quality. The correlation flanked by housing and non-housing expenditure is well articulated. Considers household spending pattern and the leverage effect.	Its confusion with poverty measurement. Insensitive to the living cost of different areas. Does not account for the influence of housing quality on location preference on housing cost (no account of location tradeoffs). Creates a certain threshold above which affordability becomes increasingly subjective. Focuses on the economic dimension of affordability. Requires an element of generalization and judgment about household type.	Commuting cost. Non-housing related expenditure. Geographic location. Household size. Loan amount. Household Composition. Spending Pattern.	Discerning norms for mortgage loans and housing allowances. For comparing HA situations of two households. Valuable in forecasting the expenditure patterns of low and medium income families. Effective in affordability studies of small areas.
	Composite Method	More sensitive to the ability of households to confront their housing and non-housing expenses, as compared to both ratio and residual income approaches. Addresses the concept of opportunity cost, that is perceived as the fundamental nature of HA, i.e., the tradeoffs that are made to acquire housing and if such tradeoffs are rational or extreme.	‘Costs of living’ are determined by some form of normative assessment. Fails to address other important issues, such as; the return on investment for housing expenditure, in terms of neighborhood and housing quality. Insensitive to the tradeoffs between cheap and affordable housing. Excludes household savings, wealth and other financial aids while depending entirely on household income. Superficially defines the point at which individuals’ acquires the right to live independently.	Living Standard, neighborhood quality. Running costs Maintenance costs, and commuting costs. Rental or Mortgage payments. Externalities: Cost saving on transport, living styles	In measuring rental housing affordability. Affordability trends over time of a region.
Scarcely Used Approaches	Behavioral	Considered to be more accurate in demonstrating the households’ expenditure pattern. Integration into the normative approach is possible, for determining the benchmark affordability ratios.	Difficult to access data required for its assessment. Sometimes shows a vague proof on the behavioral pattern of people’s housing consumption.	Household income. Household characteristics. Housing Choice.	Forecasting household consumption patterns and choice behavior.
	Subjective	Enables respondents to reveal various scenarios and issues that shape their housing stability, which are rarely measured. Accounts for differences of what informs housing choice, like taste and experience.	Fluctuates over time more often than objective measures. These indicators are often derived from samples that do not represent the entire population. Therefore, the generalizability of findings could be limited.	Household income. Household Composition. Household Perception. Housing Quality. Housing Type.	Assessing housing consumption patterns. Forecasting real estate prices. Mortgage/loan repayment ability of home owners and rental housing.
Emerging Innovative Approaches	MCDM	Aids in network and complex decision making Offers a means of problem structuring and working through information. Without all measures being converted into the same unit; social, cultural, economic and environmental considerations can be traded off.	Time consuming with large numbers. Ignores the different effects among clusters. Perfect consistency is very difficult. Some forms of MCDM may be deterministic. Does not take into account the uncertainty in weightings. Different models of MCDM can provide dissimilar outcome if used for the same problem.	Housing Price. Household Income. Externalities: Cost saving on transport, living styles, etc.	Assessing sustainable housing affordability.
	Gini Coefficient	Easy interpretability, since it is founded on ratio analysis. Considers segmentation and examines inequality in HA. Allows the comparison of income distribution in different countries, regions over time.	As a relative measure, its use and interpretation is controversial. Neglects the causes of inequality. Data on absolute regional and individual income is lost.	Household net income. Monthly rent	Estimating the relationship between income inequality and housing affordability of a given population.
	MPP	Permits a better exploration of housing price dynamics. Can offer salient information on future evolution trends of housing prices. Enables the comparison of the effectiveness of housing policy on price trends in various housing units’ sizes across cities over time. It is absolute data-driven, and no assumptions are imposed on the model.	The three-dimensional plot and the contour map provide a lot to important information on the distribution dynamics, however, they are difficult to interpret. The three-dimensional plot and the contour map provides a lot of important information on the distribution dynamics, however, they are difficult to interpret. The contour maps and three-dimensional plot provide much salient information on distribution dynamics, but the interpretation is very difficult and challenging.	House Price. Household Income.	Regional comparison of housing affordability trends.

#### Conventional approaches

There are three kinds of conventional approaches as identified in literature. These are Income ratio method (IRM), residual income method (RIM) and composite method (CM). However, IRM and RIM are generally classified as normative measures. It refers to the certain threshold value of a standard or a limit of HA [11]. This implies that a list of benchmarks are set to determine if a given household income can offset housing cost. Normative measures have been dominant and frequently utilized in housing affordability research.

**Income ratio method (IRM)**

The IRM designates a threshold value or percentile level of housing cost to income ratio in assessing the housing consumption ability of households [49]. It assumes that no matter the household income, a certain percentage of their income will always be devoted to housing-related expenses. This offers a method that enables researchers, to set a benchmark for HA based on empirical data analysis. The IRM has a long history of development and are of several types such as: housing-expenditure-to-income-ratio, rent-to-income-ratio, ongoing-housing-cost-to-income, house-price-to-income-ratio, housing-loan-repayment-to-income, debt-to-housing-price and mortgage-to-income.

Usually, new IRM is coined to address the weaknesses in an older method. For instance, Wegmann, [9] modified IRM and coined a replacement metric called the subsidy per housing affordability equivalent (SHARE) ratio. Nonetheless, traditional approach of IRM is simply the same as the definition. That is, households suffer HA stress when the ratio of income to housing cost (the affordability ratio) go beyond a given threshold ratio. Mathematically, IRM states that:

$$Housing\;Affordability=\frac{House\;Price}{Income\;of\;the\;Household}$$ (1)

The most common of this method is the Price to Income Ratio (PIR) method. It refers to the ratio of median house price and median annual household income. In essence, it measures the number of years that a household needs to accumulate their wealth from their disposable annual incomes in order to purchase an average housing unit:

$$\mathrm{PIR}=\frac{Average\;Unit\;Price\;of\;Housing\times House\;Size}{Per\;Capita\;Annual\;Disposable\;Income\;per\;Household\times Population\;per\;Household}$$

HA=f(Price,Long−termIncome,GovernmentPolicy).

This model is highly associated with Paldam Macau’s (1970) equilibrium model which states that:

$$D(P,Y_{o},G_{o})-Q^{d}=0$$

$$S(P)-Q^{s}=0$$

$$Q^{d}=Q^{s}$$

Where, the demand for residential properties (Qd) is a function of price (P), the long-termed income (Yo) and the government policy (Go). The supply of residential properties (Qs) is a function of price (P).

***Use of IRM*:** IRM has often been used due to its relative ease of application. For instance, to determine social housing qualification, and in estimating the size of groups with affordability issue, as well as to assess the prospective borrowers’ ability to service a mortgage. Dong and Zhou, [5] used the ratio of rent to income (RIR), to empirically analyze the impact of HA on the permanent migration will of rural-urban migrants. Similarly, Rowley, et al. [50] examined how the use of ratio measures could give consideration to broader financial stress.

***Weaknesses in IRM*:** IRM suffers multiplicity of weaknesses like the inability to consider quality changes over time of housing stock, and the influence of housing cost appreciation [51]. It designates a small value for a certain cordon of income to housing cost [21] and erroneously assumes that households with different income can afford all non-housing expenditures with 70 percent of their income. IRM is unable to estimate the amount of new households that should have emanated if individuals did not share houses or had remained in their parents’ houses as a result of their inability to afford rental or mortgage payments [52,53,11].

Most prominently, the ratio measures generally do not consider the spatial dimensions of transport cost, irrespective of the enormous effect housing location exerts on household commuting expenditure [14]. Hence, underestimates the number of households over burdened by combined commuting and housing costs. Fundamentally, IRM focuses on the “typical” (median) property and ‘typical’ (median or average) household; hence it neglects the price and property type variations which the first home buyers as well as those low and moderate income households would preferably demand. It can also underestimate the affordability burden of lower-income households and could overestimate such burden for households with higher income. Consequently, its adoption as a measure of HA has always faced criticism.

**Residual income method (RIM)**

RIM was developed as a better alternative to IRM. It perceives HA from a basic non-housing consumption perspective. Thus, it reveals the interaction between housing cost, income and non-housing expenditure [54]. RIM is hinged on the idea that HA is the ability of households to offset their housing cost, yet retain the capacity to meet non-housing expenditures [11]. Simply put, the income left after housing payment. It is the belief of the protagonists of this method that it makes more sense to evaluate if households posses enough income to meet other none housing expenses after offsetting housing cost, instead of assessing an arbitrary percentage of income consumed by housing [54]. Relying on this notion the “shelter poverty” concept was introduced. It describes as “shelter poor” households whom having paid for decent housing, becomes incapable of meeting other non-housing needs at a minimum socially acceptable standard [29]. A phenomena described as ‘after housing’ poverty, in poverty literature. This implies that RIM cares whether the income left after housing expenses plummet beyond shelter poverty or housing-induced poverty levels [53]. Recently, Luckey [10] introduced a location-sensitive residual income (LSRI) technique, which considers the benefits and impacts of transportation decision on HA. However, mathematically RIM is given generally by:

HousingAffordability(HA)=f(income,Housingcosts,expenditureonnon−housingnecessities)

Suchthat:HA=f(Inc,HC,NHExp) (2)

Hence, RIM posits that expenditure on one item would mean the potential sacrifice of others [11]

RIM = residual income − housing costs

= (Household disposable income–basic non-housing living expenses)–monthly mortgage repayments:

When RIM is less than 0, monthly disposable income of the household after meeting the basic living expenses is not enough for mortgage loan repayment, and vice versa [55].

***Use of RIM*:** Yang, et al., [56] used this method in Beijing to analyze the relationship between affordable housing programs and household accessibility to public services and affordability of decent housing. Revington and Townsend, [57] identified affordable rental housing locations when rapid public transit services are considered using RIM. More recently, Mundt, [58] applied a comprehensive RIM where affordability is at risk to determine household types and market segments.

***Weaknesses in RIM*:** Yip, [59] allergies its confusion with poverty measurement, while Henman and Jones, [60] claim that a separate benchmark is required for each household rather than a uniform percentage, This implies that in solving problems of HA, data requirement transcends housing and labor markets, thus, making it more burdensome. RIM like the IRM is economically focused, and as a result do not assess the non-monetary and qualitative aspects of housing. It problematizes affordability study in the sense that it relies on the minimum housing and non-housing consumption, which is considered subjective and differs among different classes of income, regions and cities, and therefore, should not be used in the analysis of HA on a macro level.

**Composite method (CM)**

As the debate over the methodological superiority of the RIM over IRM rages, other studies seem to favor CM [61]. Indeed, arguments have been raised by earlier studies that it is not possible to account for all the concerns associated with HA in one simple measurement approach. The protagonists of CM maintain that HA should not be assessed using one approach [62] but combining a number of concepts [63] and developing multiple overlapping measures [64] that can provide better understanding of HA which is more reliable; and reduces the methodological weaknesses in IRM and RIM.

Generally, this method uses the composite regression equation as specified thus:

$$Y∼N(\theta ,\sigma^{2})$$ (3)

Where, Y is the Dependent (or Response) variable; *θ* is the constant or intercept of the regression model, *σ*^2^ is the variances and covariances of the random term. Hence, the model can be written as:

$$Y_{ij}=\beta_{o}+\beta_{1}X_{1ij}+\beta_{2}X_{2ij}+\beta_{3}X_{3ij}+\mu_{ij}$$ (4)

Such that,

$$Y_{ij}=\left[\begin{array}{c} Y_{i,1}\\ Y_{i,2}\\ Y_{i,3}\\ \vdots \\ Y_{i,k} \end{array}\right];\;\beta_{o}=\left[\begin{array}{c} \beta_{oj,1}\\ \beta_{oj,2}\\ \beta_{oj,3}\\ \vdots \\ \beta_{oj,k} \end{array}\right];\;\beta_{j}=\left[\begin{array}{c} \beta_{j,1}\\ \beta_{j,2}\\ \beta_{j,3}\\ \vdots \\ \beta_{j,k} \end{array}\right];\;and\;\mu =\left[\begin{array}{c} \mu_{i,1}\\ \mu_{i,2}\\ \mu_{i,3}\\ \vdots \\ \mu_{i,k} \end{array}\right]$$

*μ* = Error associated with the model.

Y is the estimator for affordability while X_1_, X_2_, and X_3_, are identified housing challenges (factors) in the study area; $\beta_{j}^{\prime s}$ and coefficients of $X_{i}^{\prime s}$ in the model.

***Use of Composite Method*:** Thalmann, [65] combined the three indicators of affordability: rent-to-income ratio, housing consumption and quality based measures. Updating his studies in Thalmann, [22], the author used the RIM rather than the rent-to-income ratio (RIR) and computed it along with housing consumption measure and quality-based measure to quantify and determine over consumption and overpayments of housing services. Ndubueze, [62] refined and updated in Ndubueze, [66] combined the RIM and PIR while adjusting for housing quality to formulate an aggregate measure of HA. Recently, Tang, [64] used RIM (budget and poverty‐line standard), along with RIR in order to complement their weaknesses and optimize their strengths for rental affordability measurement. Similar to Sunega and Lux [67], Sarı and Khurami, [42] assessed HA using a composite of subjective approach, RIM and IRM.

***Weaknesses in CM*:** It considers household income as the only financial source of housing purchase. But other distortion factors such as family savings, housing subsidies and other capital incomes are neglected. CM does not address what gets back to households for what is spent on housing in terms of neighborhood and housing quality [68]. CM produces misleading result when presented as an independent measurement unit since it identifies good or bad policy, but fails to provide how the policy can be improved. In application, the composite is faced with challenges of which indicators should be used to devise the composite in the first place? What should the individual influence of each sub-indicator on the composite be? Are the data sources comparable when measuring the same phenomenon in a cross-country perspective? These weaknesses must be considered before applying the technique.

#### Scarcely used measurement approaches

**Behavioral approach**

The behavioral approach assesses affordability by examining the housing decisions of individual households. It simply reviews the housing consumption behavior of a household. This implies that the behavioral approach focuses on the normal housing decisions while adjusting for what households with certain incomes and compositions confronted by certain prices opt to pay for housing [19]. Behavioral approach deals with understanding the choices households make regarding location, type, tenure, and size of housing. It also deals with the problem of mortgage arrears and repossessions, so as to investigate the household’s affordability based on their decision. Households housing consumption behavior is a veritable tool for HA assessment. However, the outcome of the research performed by Fein, et al., [69] and Maclennan, et al., [70] were inconclusive. Yet, the advocates of behavioral approach believe that when HA problem is assessed as an explicit issue and the empirical data available is sufficient to support an in-depth research, this approach would better reveal households’ spending pattern towards developing indicators of HA [59]. It is also believed that the threshold developed from a behavioral point of view, that is, the point at which the increments in cost of housing indicates a different qualitative model in its interaction with household income, suggests an indication of housing in-affordability, and can be employed as well to validate affordability outcomes of other measurement approaches.

***Use of Behavioral Approach*:** Arguably, the behavioral approach can be integrated into the normative measures in determining the threshold affordability ratios. Using this approach, Wood et al., [71] observed that the behavioral effects of several kinds of housing allowances are insufficiently examined, because housing subsidies could discourage unemployed households from seeking employment opportunities in other locations. Similarly, Grinstein-Weiss, et al., [72] observed that low income households saving behaviors are mainly influenced by the structured opportunities they are offered. Recently, Olanrewaju and Woon, [73] combined both internal and external criteria that influence household decision-making to study homebuyer/homeowner behaviors.

***Weaknesses in the Behavioral Approach*:** The behavioral approach is considered difficult to compute and many a time unable to present a real evidence of the behavioral path of people's housing consumption. However, with the invention of behavioral economics and the perceived weaknesses in normative measures, behavioral approach is believed to have potential for further development.

**Subjective approach**

Given that both behavioral and normative measures are regarded as objective measurements. Kearns et al., [74] offered a completely different approach, called the subjective approach. Subjective approach summarizes the subjective evaluation of households’ perception of their housing need [75] in relation to housing quality and condition, affordability dilemma and overcrowding. It directly asks respondents if they consider their house affordable or not, as the qualitative and subjective measurements are checked against their financial position and other quantitative criteria. The data generated are used to establish a threshold cordon of their HA with the conviction that households are better positioned to offer the best assessment of their housing situation. This approach rests on the assumption of *homo economicus*. Following this approach HA is a function of household income. Mathematically, it states that,

HousingAffordability=f(Householdincome) (5)

***Use of Subjective Approach*:** Subjective indicators are often employed as predictors controlling price of property [75] or as a complement for objective indicators. Stiglitz, et al. [76] noted that obvious difference between objective and subjective indicators which is impossible to explain under human psychology or money illusion could lead to ineffective distribution of public resources and weakening trust in official statistics, in some countries (e.g. the UK). Nevertheless, it is believed that subjective measures can offer other information left out under standard objective measures, and can support cost-benefit analysis and policy evaluation as well as aid the identification of potential policy problems [77]. It is therefore remarkable, that studies in housing have marginally devoted interest in developing alternative HA measures that responds more precisely to subjective perceptions of housing stress [68].

***Weaknesses in the Subjective Approach*:** Subjective indicators easily fluctuate over time more than the objective measures [42]. This approach requires normative assessments of what represents the “living costs”. However, it seems that adopting some sort of normative basis for definition and measurement is unavoidable in any HA analysis [78].

#### Emerging innovative methodologies

New methods of measuring housing affordability are emerging as a result of improved understanding of affordability burden, and the fact that the existing HA measures focus mainly on the fiscal dimension while other modified approaches mostly re-emphasized the weaknesses of the methods they tried to amend. Three emerging novel methods (which are essentially adaptations from the distinctive measurement approaches, but with more robust methodologies) were identified in the literature. Examples are; multiple criteria decision making (MCDM); Gini coefficient and mobility probability plot (MPP) methods.

**Multiple criteria decision making (MCDM) method**

MCDM is a tool involving both qualitative and quantitative factors [79]. It is a set of methods that supports the consideration and aggregation of several criteria, often numerous, mostly at variance, decision criteria [80], which is used to describe, choose, rank or sort, a range of alternatives to support a decision process [79].

***Use of MCDM*:** Several models within the MCDM methodological framework have been suggested [81]. The most commonly used MCDM models include; the Complex Proportional Assessment (COPRAS), Analytical Hierarchy Process (AHP) and Technique for Order of Preference by Similarity to Ideal Solution (TOPSIS) [7]. A novel study by Mulliner, [68] refined and updated in Mulliner, et al., [6] adapted the COPRAS model to assess HA, as an instance on how to apply MCDM for HA analysis. The findings indicate that taking into account additional criteria that clearly consider housing location and community sustainability, housing quality amongst others; against mere economic considerations alone, can significantly impact affordability calculations. Adopting the same method in Sabah, Malaysia Said, et al., [80] assessed the best locations for the development of sustainable affordable housing programs. Mulliner, et al., [7] investigated the applicability of several models within the MCDM methodological framework, for optimal HA assessment. The study revealed that various MCDM models can be applied for sustainable HA assessment, as a result of the model's ability to address the numerous conflicting decision criteria and the multidimensionality of issues found in HA problems. Mathematically put -

HousingAffordability=f(Alternatives)intheirrankingorder.Suchthat:

$$HA=\beta_{i};\;i=1,\;2,\ldots ,n$$ (6)

For instance, the COPRAS method is represented as

$$d_{ij}=\frac{q_{i}}{\sum_{j=1}^{n}x_{ij}}x_{ij}$$ (7)

where *x*_*ij*_ is the value of the i-th criterion of the j-th alternative, and qi is the weight of the i-th criterion.

***Weaknesses in the MCDM*:** When several models within the MCDM methodological framework are employed to assess same problem, they often generate different results [81]. MCDM is susceptible to manipulation which could alter the degree of result accuracy. Daniel, et al., [82] remarked that though the complex nature of this measure deepens the overall understanding of multiple concerns which breed HA problem, there is a chance that its complexity could weaken the uptake by analysts and researchers.

**Gini coefficient method**

The Gini coefficient measures the inequality within the income distribution of a given population (by means of a ratio analysis). It captures the influence of income inequality in estimating HA. Various approaches are applicable here, for instance, RIR estimate based on average. However, the method is based on net PIR. Mathematically,

$$GH=\frac{Household\;Net\;income}{Housing\;Price}$$

Such that the HA at a period t, can be estimated as:

$${GH}_{t}=\beta_{o}+\beta_{1}{GA}_{t}+\beta_{2}{\left(\frac{P}{A}\right)}_{t}+{\bar{\beta }}_{3}CONTROLS+\varepsilon_{t}$$ (8)

where, t is a time period index, GA and GH are net income and housing affordability Gini coefficients, respectively, $\frac{P}{A}$ is the average housing price-to-net income ratio (across all households at time t), CONTROLS is a matrix of macroeconomic variables, including gross domestic product (GDP), unemployment rate (UNEPR), exchange rate (EXR), among others. Meanwhile, *β*_*o*_ *to β*_2_ are parameters, ${\bar{\beta }}_{3}$ is a vector of parameters and *ε*_*t*_ is a random disturbance term associated with the model.

***Use of Gini Coefficient Method*:** The Gini coefficient method can be applied in the comparison of income distributions across different sub-populations [37]. A novel study by Ben-Shahar and Warszawski, [83] adapted the Gini coefficient method to estimate income inequality in HA, and to evaluate the factors associated with the time-varying inequality measures of HA. More recently Dong, [37] used this method to evaluate the effect of growing income inequality on low income tenant families’ worsening rental affordability.

***Weaknesses in Gini Coefficient Method*:** The Gini coefficient mainly lies on its relative nature; then information on absolute regional and individual income is lost, and fails to consider the causes of inequality [84]. Also, as a measure of inequality and the fact that it only isolates the inequality in the distribution of a particular macroeconomic variable. Thus, it isolates the magnitude of the variable to a relative rather than an absolute degree which implies that a country can witness simultaneously, for instance a rising income inequality and a reduction in absolute poverty [84]. It is also quite possible to have countries of disparate income levels having similar or even in some cases, identical Gini coefficients, which implies a high income country having the same Gini coefficient as a very low income country, or moreover a region with predominantly low income and low quality housing, having the same HA inequality index as a region with higher income and predominantly higher housing quality. Consequently, Gini coefficients should not be used as a stand-alone quantitative measure of a particular variable such as wealth and housing affordability. Instead, it is advisable to augment it with other measures that capture the variable on an absolute scale.

**Mobility probability plot (MPP)**

MPP was originated by Cheong and Wu [85] for studying regional inequality. It is based on PIR which is obtained by dividing the house price by income [8]. MPP is the transitional dynamics of HA based on Markov transition matrix approach and the stochastic kernel technique. It is however used to analyze the mobility of PIR for cities, thereby measuring city-level trends of HA. MPP is expressed in percentages (ranges between −100 and +100). A positive value of MPP suggests that the city will have a net probability of moving upward in the distribution of PIR, thereby indicating that the PIR of the city will become higher and higher, and the housing price will be more unaffordable. In contrast, a negative value of MPP indicates that the city has a net probability of moving downward in the distribution of PIR which indicates that the city has a high tendency of registering a decline in the PIR.

MPP can be computed by calculating *p*(*x*) as:

$$p(x)=\int_{x}^{\infty }g_{r}(z/x)dz-\int_{0}^{x}g_{r}(z/x)dz$$ (9)

Where, ∫ *is the function of integration*, X is an observed value of relative household income at time t; and *g*_*r*_(*z*/*x*) is the transition probability kernel which maps the distribution from time t

***Use of MPP*:** A novel study by Cheong and Li, [8] adopted the MPP to study the transitional dynamics of HA indicators. Li, et al., [40] employed the MPP method to analyze the mobility of housing price growth and impact the enactment and withdrawal of home purchase restrictions (HPR) policy have on changes in housing price.

***Weaknesses in MPP*:** Li, et al., [40] remarked that although the contour maps and three-dimensional plot generated provides much salient information on the distribution dynamics, the interpretation may be very difficult and challenging.

## Discussion

The literature findings revealed several conceptual irregularities of HA definitions which exists in most references included in this study. However, whether explicitly or implicitly defined, or formulated methodically on an operational basis, such as, loan/rent/mortgage to income ratio, the vital constituents of the definitions like monthly rent or gross monthly income differ considerably, depending on the context, research objectives and data available. An accurate definition and measurement of HA would inform appropriate policy intervention, but a narrowly construed definition would promote other agendas with little interest in affordability.

Furthermore, several HAMA have been developed by researchers, and applied in varied situations over the last few decades building on past research findings, in an attempt to influence policy responses more precisely. Some of these approaches like the ratio measures are often utilized due to their ease of computation, and appeal to peoples’ common sense experience; since they generally require data on housing cost and income. However, some others are underutilized due to their high subjectivity demand or complexity. It is notable that, except for the emerging innovative approaches, many of the other measures do not consider household size, transportation cost, housing choice and household preference. They erroneously assume that there are no distinctions between household and household characteristics. In addition, most approaches experienced similar nature of development and modification, for instance, the transition of the income ratio measures from housing expenditure and income ratio to loan/mortgage/rent and income ratios; as well as the innovation of residual measure, amongst others. In recent years, due to the observed weaknesses of prevalent approaches, researchers began combining different methods of HAMA. It was also observed from literature that in identifying problems of affordability, the results of several HAMA are weakly correlated when applied independently to the same problem of same time frame. But a strong level of congruity is reported on affordability results when they are combined. This combination of multiple approaches is intended to check the weaknesses that are preponderant in certain approaches, especially in normative measures and to further enhance the reliability of measurement outcomes.

It is believed that the modified approaches, along with some approaches in their original form, can reach extreme heights of success in their application (e.g. ratio, residual and subjective measure combinations, amongst others), if proper evaluations of their strengths alongside their weaknesses are conducted. More so, the recent realization by researchers for the need to consider the multiple dimensions of affordability stress, such as social and ecological dimensions as well as economic dimension have allowed more complex (innovative) methods to emerge and earlier ones, modified. Thus, offering new insights into the HA concept. As summarized and presented in Table 3, this study identifies the commonly and scarcely used as well as the emerging innovative approaches for measuring HA. It also determines their applicability in a particular circumstance by examining which strengths and weaknesses that are prevalent in each approach. Other range of issues involved in undertaking HA assessment was expounded upon, in order to avail researchers with multiple choice of several types and caveats of concern to the validity of measurement approaches for solving specific affordability burden, housing stress and calculating areas for affordable housing development. However, the determination of the most appropriate affordability measurement approach could be influenced by the description of research objectives, the orientation of the researcher, policy guidelines and available data. The assessment of HAMA performed in this review offers a framework on how these approaches could be applied in specific circumstances.

In summary, this paper is considered as an attempt for the generation of further and evolving discussions within HA research domain, which would ultimately lead to a clearer and more holistic insight into the dynamic nature of HA. It could also inspire a renewed research agenda for conceptual refinement and the development of more assessment methods that can draw closer links with sustainability principles by taking into account the social, environmental and economic criteria that impact on the quality of life of households.

### Suggestions for methodological improvement of selected HAMA

Many researchers of diverse orientation have put forward several recommendations and suggestions on how different HAMA can be enhanced, in order to eradicate their various inherent weaknesses. Some of the key recommendations as suggested by housing researchers are presented in Table 4.

**Table 4:** Summary of the suggestions for the improvement of selected HAMA.

Key Reference	Approach/Method	Suggestion/Recommendations
[86,12,6,7,64,82]	Housing Affordability Concept	In conceptualizing HA, the social, economic and ecological aspects that determine households’ well being must be considered. Broadening the concept of housing affordability measure to incorporate material deprivation, would capture individuals truly having HA problems (not merely individuals at risk).
[21,22,68,87,3,50,88,53]	Normative Measures (IRM and RIM)	Apply equivalent income in place of income, while adjusting household incomes for household composition. This modification could reclassify more single person households that were wrongly classified as low income. The ratio measure can be improved by considering the experiences of individuals over time (i.e. use of longitudinal data). Especially, with regards to the negative and positive life circumstances that breed HA stress. Use of subjective approach to improve the normative measures. However, this suggestion underestimates the role households’ subjective assessment play in comprehensive understanding of the HA problems. Formulate objective indicators to consider households subjective perception of problems of housing. This may offer a more precise approach of measuring housing needs. To improve the IRM, compare poverty lines with housing cost deducted income. Also provide data disaggregated by household type and develop many ratio measures for different types of household. Use residual income or disposable income against housing cost rather than overall household income for it better captures housing cost induced poverty. Subjective evidence of material hardship and payment problems could be employed to validate ratio measures and identifies the best thresholds to apply.
[65,64,82,62]	Composite Method	Develop multiple overlapping measures of affordability. Take into account all qualitative and quantitative criteria that affect household wellbeing. Material deprivation measure if applied in combination with the 30/40 ratio, offers a more precise measure of poor HA experience which is naturally associated with wider concerns in which these issues are suffered.
[42,89,90]	Subjective Approach	Use of a scale, while collecting housing cost burden data as reported by households, instead of reducing it into categories. Case study may provide more support for developing the subjective approach.

### Suggestions for future research

There is a dearth of research focus comparing HA issues between developing and developed economies, and the appropriateness of applying normative measures in developing economies. Almost no study in the last decade compared the distinctive approaches of affordability except partly for [67] and [42], who compared and/or combined the objective and subjective affordability measures. In addition, this review affirms the dominance of the IRM despite the overwhelming weaknesses except for RIM in a few quarters. Other measures are yet to gain traction amid academics, researchers and analysts. The remote and immediate causes of these disparities beg for attention. More studies are encouraged to utilize the emerging methodologies advocated by this review in different regions and context for a revalidation of the benefits attributed to it. Furthermore, future studies are encouraged to research on newer approaches that can utilize and incorporate the strengths of other approaches, while addressing or altogether eradicating their weaknesses.

## Conclusion

HA problem is a crucial issue affecting several cities across the globe in both developing and developed countries. Measuring HA dates back more than 40 years, since 1970’s. During this timeline a huge number of approaches were developed by researchers of diverse orientation, leading to what has been referred to as ‘methodological chaos’ and according to Wilcox (1999) a ‘vexed’ concept (as cited in [91]). This is reflected in the fact that HA literature reveals an abundance of differences and at times contradictory definitions, concepts, techniques and methodological ideas about HA. Therefore, this review recommends that the immediate need for the future of HA literature is amongst other things to resolve the confusion over ‘the definitions/concepts and measurement approaches/methods/techniques of HA analysis. This review makes a beginning at this need by tracing the origin, theoretical underpinnings and growth of HAMA; as well as the subsequent evolution of the various methodologies. A classification of the methodologies into three main approaches is provided and the salient characteristics (weaknesses and relative strengths) of these approaches are compared and contrasted.

This review revealed the lack of consensus on the most appropriate approach. However, the best method can be obtained by analyzing the various weaknesses and strengths inherent in each method. Researchers and policy makers must be detailed about which HAMA they adopt, why they adopt it, and if the method being adopted is appropriate for its purpose. The study then calls for continued conceptual refinement and further development of more appropriate approaches that could better consider the multi-dimensionality inherent in HA problem. The issues discussed in this paper will assist in formulating techniques that can be used in measuring HA in a sustainable manner. Measuring HA transcends housing price and income terms. Therefore, an ideal HA metric must take into consideration a range of social, environmental and economic criteria; which borders on broader concept of housing appropriateness covering accessibility, affordability, amenity and adequacy, that impact on residents’ quality of life. This will ensure that both sustainability and affordability concerns are tackled concurrently in any HA analysis. It is hoped that this research will inspire future studies to establish a broader housing affordability concept and metric that is better aligned with sustainability.

Since MCDM method fairly takes into account the dynamism of HA indicators, which addresses the major measurement weaknesses in the conventional approaches. Though there are insufficient studies that employ the MCDM method due to its complexity, reporting technique and heterogeneity. It may be an effective and efficient method of measuring HA problem. Therefore, to effectively explore the potential benefits and validate the soundness of this emerging novel method, future studies and policy makers are encouraged to utilize it.

### Research contribution

This study makes four contributions to the international HA literature. Firstly, it developed a classification scheme of HAMA that is based on the frequency of application and developmental trends. Secondly, it structurally reviewed existing literature to guide the research on HA concept and measurement. Thirdly, it identified several approaches and weaknesses of HAMA; as well as suggested techniques that can be effectively used to improve different HAMA. Again, it identified issues of interest to be undertaken by future studies. This study will be a guide for improving HAMA, as well as aid policy makers in shaping policy framings and informing on appropriate housing policy direction. Finally, this study satisfies early-career researchers’ need for an easy reference to HAMA studies and publications.

## Supporting information

S1 Table Review design. (PDF)

S2 Table Extraction sheet for source type and database sources included in the study. (PDF)

## References

[1] Gabriel S, Painter G. Why affordability matters. Regional Science and Urban Economics. 2018 7 6 (In Press). 10.1016/j.regsciurbeco.2018.07.001

[2] Taltavull P, Juárez F. Housing affordability. A literature review. Galega Magazine of Economics, 2012;21 (2), 1–24. Available at https://goo.gl/23Kocj

[3] Jewkes M, Delgadillo L. Weaknesses of housing affordability indices used by practitioners. Journal of Financial Counseling and Planning. 2010;21(1).21, 43–52.

[4] Arman M, Zuo J, Wilson L, Zillante G, Pullen S. Challenges of responding to sustainability with implications for affordable housing. Ecological Economics. 2009 10 15;68(12):3034–41. 10.1016/j.ecolecon.2009.07.007

[5] Dong X, Zhou W. Housing Affordability and Permanent Migration Intention of Rural-Urban Migrants. Chinese Journal of Urban and Environmental Studies. 2016 6;4(02):1650019.

[6] Mulliner E, Smallbone K, Maliene V. An assessment of sustainable housing affordability using a multiple criteria decision making method. Omega. 2013 4 1;41(2):270–9. 10.1016/j.omega.2012.05.002

[7] Mulliner E, Malys N, Maliene V. Comparative analysis of MCDM methods for the assessment of sustainable housing affordability. Omega. 2016 3 1;59:146–56. 10.1016/j.omega.2015.05.013

[8] Cheong TS, Li J. Transitional distribution dynamics of housing affordability in Australia, Canada and USA. International Journal of Housing Markets and Analysis. 2018 2 5;11(1):204–22. 10.1108/IJHMA-01-2017-0003

[9] Wegmann J. Measuring What Matters: A Call for a Meaningful Metric of Affordable Rental Housing Production Cost-Efficiency. Housing Policy Debate. 2014 10 2;24(4):692–716. 10.1080/10511482.2014.944851

[10] Luckey KS. Affordable for whom? Introducing an improved measure for assessing impacts of transportation decisions on housing affordability for households with limited means. Research in Transportation Business & Management. 2018 12 1;29:37–49. Do: 10.1016/j.rtbm.2018.04.003

[11] Stone ME. What is housing affordability? The case for the residual income approach. Housing policy debate. 2006 1 1;17(1):151–84. 10.1080/10511482.2006.9521564

[12] Nwuba CC, Kalu IU. Measuring housing affordability: the two approaches. ATBU Journal of Environmental Technology. 2018;11(1):127–43. Available at https://www.ajol.info/index.php/atbu/article/view/177577/166927

[13] Mulliner E, Maliene V. An analysis of professional perceptions of criteria contributing to sustainable housing affordability. Sustainability. 2015 1;7(1):248–70. 10.3390/su7010248

[14] Mattingly K, Morrissey J. Housing and transport expenditure: Socio-spatial indicators of affordability in Auckland. Cities. 2014 6 1;38:69–83. 10.1016/j.cities.2014.01.004

[15] Aribigbola A. Housing affordability as a factor in the creation of sustainable environment in developing world: the example of Akure, Nigeria. Journal of Human Ecology. 2011 8 1;35(2):121–31. 10.1080/09709274.2011.11906397

[16] Makinde OO. Housing delivery system, need and demand. Environment, Development and Sustainability. 2014 2 1;16(1):49–69. 10.1007/s10668-013-9474-9

[17] Howenstine E. J. Attacking housing costs: foreign policies and strategies. New Brunswick, N.J.: Center for Urban Policy and Research, 1983; 133p.

[18] Maclennan D, Williams R. Affordable housing in Britain and America. Joseph Rowntree Foundation, York 1990.

[19] Bramley G. An affordability crisis in British housing: dimensions, causes and policy impact. Housing Studies. 1994 1 1;9(1):103–24. 10.1080/02673039408720777

[20] Whitehead CM. From need to affordability: an analysis of UK housing objectives. Urban Studies. 1991 12;28(6):871–87. 10.1080/00420989120081101

[21] Hancock KE. 'Can Pay? Won't Pay?'or Economic Principles of'Affordability'. Urban studies. 1993 2;30(1):127–45. 10.1080/00420989320080081

[22] Thalmann P. ‘House poor’or simply ‘poor’?. Journal of Housing Economics. 2003 12 1;12(4):291–317. 10.1016/j.jhe.2003.09.004

[23] Burke T., Ralston L. Measuring housing affordability. AHURI Research and Policy Bulletin No. 45, Australian Housing and Urban Research Institute Limited, Melbourne 2004 Retrieved from https://goo.gl/TaS8Ei

[24] Leishman C, Rowley S. Affordable Housing In The Sage Handbook of Housing Studies, edited by Clapham D. F., Clark W. A. V., and Gibb K., 379–396. Los Angeles, CA: Sage 2012 Available at https://goo.gl/4RAEBk

[25] Minchenko MM, Nozdrina NN. The dynamics of housing affordability for the population of Russia in 2008–2014. Studies on Russian Economic Development. 2017 3 1;28(2):191–203. 10.1134/S1075700717020071

[26] Chapman P. Housing affordability in Australia, Research and Policy Bulletin, 2 2006.

[27] Rowley S, Ong R. Housing Affordability, Housing Stress and Household Wellbeing in Australia. Final Report No. 192; Australian Housing and Urban Research Institute: Melbourne, Australia. 2012. Retrieved from https://goo.gl/hEbGga

[28] Quigley JM, Raphael S. Is housing unaffordable? Why isn't it more affordable?. Journal of Economic Perspectives. 2004 3;18(1):191–214. 10.1257/089533004773563494

[29] Stone ME. Shelter Poverty: New Ideas on Housing Affordability; Temple University Press: Philadelphia, PA, USA 1993.

[30] Maher Ken. Housing affordability: Why architecture matters. ArchitectureAU. 2017Retrieved from https://goo.gl/FNWKaf

[31] Napoli G. Housing Affordability in Metropolitan Areas. The Application of a Combination of the Ratio Income and Residual Income Approaches to Two Case Studies in Sicily, Italy. Buildings. 2017;7(4):95 10.3390/buildings7040095

[32] Stone ME, Burke T, Ralston L. The residual income approach to housing affordability: the theory and the practice (AHURI Positioning Paper No. 139). Boston: University of Massachusetts 2011 Available at https://goo.gl/3Ze2JC

[33] Fisher LM, Pollakowski HO, Zabel J. Amenity‐based housing affordability indexes. Real Estate Economics. 2009 12;37(4):705–46. 10.1111/j.1540-6229.2009.00261.x

[34] Isalou AA, Litman T, Shahmoradi B. Testing the housing and transportation affordability index in a developing world context: A sustainability comparison of central and suburban districts in Qom, Iran. Transport policy. 2014 5 1;33:33–9. 10.1016/j.tranpol.2014.02.006

[35] Desmond M. Heavy is the house: Rent burden among the American Urban Poor. International Journal of Urban and Regional Research. 2018 1;42(1):160–70. 10.1111/1468-2427.12529

[36] HUD. Worst Case Housing Needs 2011: Report to Congress. Washington, DC: U.S. Department of Housing and Urban Development, Office of Policy Development and Research 2011 Available at http://www.huduser.org/portal/publications/progs_of_hud.html.

[37] Dong H. The impact of income inequality on rental affordability: An empirical study in large American metropolitan areas. Urban Studies. 2018 8;55(10):2106–22. 10.1177/0042098017710380

[38] Cox Wendell. What Is Middle-Income Housing Affordability? Newgeography.Com. 2018 Avaliable from https://goo.gl/Y282NB

[39] Dewilde C. Explaining the declined affordability of housing for low-income private renters across Western Europe. Urban Studies. 2018 9;55(12):2618–39. 10.1177/0042098017729077 30369643 PMC6187072

[40] Li VJ, Cheng AW, Cheong TS. Home purchase restriction and housing price: A distribution dynamics analysis. Regional Science and Urban Economics. 2017 11 1;67:1–0. 10.1016/j.regsciurbeco.2017.08.002

[41] Yang H. An Analysis of Restrictions on Housing Purchases in China. Research Paper, University of Ottawa. 2017. Available at https://goo.gl/e6fyCx

[42] Sarı ÖB, Khurami EA. Housing affordability trends and challenges in the Turkish case. Journal of Housing and the Built Environment. 2018 6 1:1–20. 10.1007/s10901-018-9617-2

[43] Nwuba CC, Kalu IU, Umeh JA. Determinants of homeownership affordability in Nigeria’s urban housing markets. International Journal of Housing Markets and Analysis. 2015 6 1;8(2):189–206. 10.1108/IJHMA-06-2014-0020

[44] Nwosa PI. Fiscal Policy and Exchange Rate Movement in Nigeria. Acta Universitatis Danubius. Œconomica, 2017;13 *(*3*)* Available at https://goo.gl/u1RDE9

[45] O’Dell W, Smith MT, White D. Weaknesses in current measures of housing needs. Housing and Society. 2004 1 1;31(1):29–40. 10.1080/08882746.2004.11430496

[46] Duffy D. A Note on Measuring the Affordability of Homeownership. Policy Discussion Forum, Quarterly Economic Commentary, ESRI. 2004 Retrieved from https://goo.gl/kLmvDt

[47] Eakes M. Preserving the American dream: Predatory lending practices and home foreclosures, Center for Responsible Lending and Center for Community Self-Help. Testimony presented at the meeting before the U.S. Senate Committee on Banking, Housing and Urban Affairs, Washington, DC 2007 Avaliable from https://goo.gl/wS4mgd

[48] Abelson P. Affordable housing: concepts and policies. Economic Papers: A journal of applied economics and policy. 2009 3;28(1):27–38. 10.1111/j.1759-3441.2009.00001.x

[49] Abeysinghe T, Gu J. Lifetime income and housing affordability in Singapore. Urban Studies. 2011 7;48(9):1875–91. 10.1177/0042098010380956

[50] Rowley S, Ong R, Haffner M. Bridging the gap between housing stress and financial stress: The case of Australia. Housing studies. 2015 4 3;30(3):473–90. 10.1080/02673037.2014.977851

[51] Linneman PD, Megbolugbe IF. Housing affordability: myth or reality?. Urban studies. 1992 5;29(3–4):369–92. 10.1080/00420989220080491

[52] Gan Q, Hill RJ. Measuring housing affordability: Looking beyond the median. Journal of Housing economics. 2009 6 1;18(2):115–25. 10.1016/j.jhe.2009.04.003

[53] Kutty NK. A new measure of housing affordability: Estimates and analytical results. Housing policy debate. 2005 1 1;16(1):113–42. 10.1080/10511482.2005.9521536

[54] Yang Z, Shen Y. The affordability of owner occupied housing in Beijing. Journal of Housing and the Built Environment. 2008 12 1;23(4):317 10.1007/s10901-008-9120-2

[55] Duan M. Investigation on housing affordability in Lanzhou, Northwest China. International Journal of Housing Markets and Analysis. 2011 5 31;4(2):180–90. 10.1108/17538271111137958

[56] Yang Z, Yi C, Zhang W, Zhang C. Affordability of housing and accessibility of public services: evaluation of housing programs in Beijing. Journal of Housing and the Built Environment. 2014 9 1;29(3):521–40. 10.1007/s10901-013-9363-4

[57] Revington N, Townsend C. Market rental housing affordability and rapid transit catchments: Application of a new measure in Canada. Housing Policy Debate. 2016 9 2;26(4–5):864–86. 10.1080/10511482.2015.1096805

[58] Mundt A. Housing benefits and minimum income schemes in Austria–an application of the residual income approach to housing affordability of welfare recipients. International Journal of Housing Policy. 2018 7 3;18(3):383–411. 10.1080/19491247.2017.1306992

[59] Yip NM. Housing affordability in England, PhD Thesis, University of York. 1995. Available at https://goo.gl/29RmfR

[60] Henman P, Jones A. Exploring the use of residual measures of housing affordability in Australia: methodologies and concepts, AHURI Final Report No.180. Melbourne: Australian Housing and Urban Research Institute. 2012. Available at https://goo.gl/bVBoEs

[61] Ndubueze OJ. Measuring housing affordability: A composite approach. In Proceedings of the ENHR 2007 International Conference Sustainable Urban Areas, Rotterdam, The Netherlands (pp. 25–28). 2007.Avaliable at https://goo.gl/X9kLPD

[62] McCord M, McIlhatton D, McGreal S. The Northern Ireland housing market and interconnections with the UK and Irish housing markets. Housing Finance International. 2011;26(1):28–34.

[63] Haffner M, Heylen K. User costs and housing expenses. Towards a more comprehensive approach to affordability. Housing Studies. 2011 6 1;26(04):593–614. 10.1080/02673037.2011.559754

[64] Taltavull P, Tang CP. Measuring the affordability of housing association rents in England: a dual approach. International Journal of Housing Markets and Analysis. 2012 7 27 10.1108/17538271211243571

[65] Thalmann P. Identifying households which need housing assistance. Urban Studies. 1999 10;36(11):1933–47. 10.1080/0042098992683

[66] Ndubueze OJ. Urban housing affordability and housing policy dilemmas in Nigeria (Doctoral dissertation, University of Birmingham). 2009. Available at https://goo.gl/po9WR9

[67] Sunega P, Lux M. Subjective perception versus objective indicators of overcrowding and housing affordability. Journal of Housing and the Built Environment. 2016 12 1;31(4):695–717. 10.1007/s10901-016-9496-3

[68] Mulliner EK. A model for the complex assessment of sustainable housing affordability (Doctoral dissertation, Liverpool John Moores University). 2012. Retrieved from https://goo.gl/dKHnv8

[69] Feins J, White JR, Charles S. The Ratio of Shelter Expenditure to Income: Definitional Issues, Typical Patterns and Historical Trends (Cambridge; MA, Abt Associates). 1977

[70] Maclennan D, Gibb K, More A. *Paying for Britain's housing* (Vol. 36). York: Joseph Rowntree Foundation 1990.

[71] Wood G, Ong R, Dockery AM. The long-run decline in employment participation for Australian public housing tenants: an investigation. Housing Studies, 2009; 24(1), 103–126. 10.1080/02673030802547421

[72] Grinstein-Weiss M, AN Chowa G, Casalotti AM. Individual development accounts for housing policy: Analysis of individual and program characteristics. Housing Studies. 2010 1 1;25(1):63–82. 10.1080/02673030903362035

[73] Olanrewaju A, Woon TC. An exploration of determinants of affordable housing choice. International Journal of Housing Markets and Analysis. 2017 10 2;10(5):703–23. 10.1108/IJHMA-11-2016-0074

[74] Kearns A., Gibb K., More A., Keoghan M., Pryce G. *Community ownership rents*: *Greater Easterhouse in the Glasgow context*. Report to the Greater Easterhouse Community Ownership Forum, Glasgow 1996.

[75] Chasco C, Gallo JL. The impact of objective and subjective measures of air quality and noise on house prices: a multilevel approach for downtown Madrid. Economic Geography. 2013 4 1;89(2):127–48. 10.1111/j.1944-8287.2012.01172.x

[76] Stiglitz J, Sen AK, Fitoussi JP. The measurement of economic performance and social progress revisited: reflections and overview. Sciences Po publications 2009–33, Sciences Po.

[77] OECD. How’s Life? at a Glance, in How’s Life?: Measuring Well-being, OECD Publishing 2013 10.1787/9789264201392-en

[78] Robinson M, Scobie GM, Hallinan B. Affordability of housing: concepts, measurement and evidence. 2006 Month, 6(04). Retrieved from https://goo.gl/72X6W5

[79] Mardani A, Jusoh A, Nor K, Khalifah Z, Zakwan N, Valipour A. Multiple criteria decision-making techniques and their applications–a review of the literature from 2000 to 2014. Economic Research-Ekonomska Istraživanja. 2015 12 20;28(1):516–71. 10.1080/1331677X.2015.1075139

[80] Said R, Majid RA, Alias A, Adnan YM, Razali MN. Sustainable housing affordability in Sabah. PLANNING MALAYSIA JOURNAL. 2016 11 10;14(5).

[81] Zavadskas E, Cavallaro F, Podvezko V, Ubarte I, Kaklauskas A. MCDM assessment of a healthy and safe built environment according to sustainable development principles: A practical neighborhood approach in Vilnius. Sustainability. 2017;9(5):702 10.3390/su9050702

[82] Daniel L, Baker E, Lester L. Measuring housing affordability stress: can deprivation capture risk made real?. Urban Policy and Research. 2018 7 3;36(3):271–86. 10.1080/08111146.2018.1460267

[83] Ben-Shahar D, Warszawski J. Inequality in housing affordability: Measurement and estimation. Urban Studies. 2016 5;53(6):1178–202. 10.1177/0042098015572529

[84] Wang XJ, Zhang JY, Shahid S, ElMahdi A, He RM, Wang XG, Ali M. Gini coefficient to assess equity in domestic water supply in the Yellow River. Mitigation and Adaptation Strategies for Global Change. 2012 1 1;17(1):65–75. 10.1007/s11027-011-9309-7

[85] Cheong TS, Wu Y. Convergence and transitional dynamics of China's industrial output: A county-level study using a new framework of distribution dynamics analysis. China Economic Review. 2018 4 1;48:125–38. 10.1016/j.chieco.2015.11.012

[86] Chen J, Hao Q, Stephens M. Assessing housing affordability in post-reform China: A case study of Shanghai. Housing Studies. 2010 11 1;25(6):877–901. 10.1080/02673037.2010.511153

[87] Gabriel, M., Jacobs, K., Arthurson, K., Burke, T., Yates, J. Conceptualising and measuring the housing affordability problem, AHURI Research Paper No. NRV3-1, Australian Housing and Urban Research Institute Limited, Melbourne. 2005. Retrieved from https://goo.gl/4Vd69y

[88] Affordability Bramley G., poverty and housing need: triangulating measures and standards. Journal of Housing and the Built Environment. 2012 6 1;27(2):133–51. 10.1007/s10901-011-9255-4

[89] Talen E. Affordability in new urbanist development: Principle, practice, and strategy. Journal of Urban Affairs. 2010 10 1;32(4):489–510. 10.1111/j.1467-9906.2010.00518.x

[90] Ruming K, Gurran N, Randolph B. Housing affordability and development contributions: New perspectives from industry and local government in New South Wales, Victoria and Queensland. Urban Policy and Research. 2011 9 1;29(3):257–74. 10.1080/08111146.2011.592136

[91] Lee CL, Reed RG. The relationship between housing market intervention for first-time buyers and house price volatility. Housing studies. 2014 11 17;29(8):1073–95. 10.1080/02673037.2014.927420

