# Supplementary material for: Methodological weaknesses in the measurement approaches and concept of housing affordability used in housing research: A qualitative study
Source: PLoS One. 2019 Aug 30;14(8):e0221246. doi: 10.1371/journal.pone.0221246 (PMC6716639; doi:10.1371/journal.pone.0221246)
Supplement: S1 Table — (PDF) [file pone.0221246.s001.pdf]

Methodological Weaknesses in the Measurement Approaches and Concept of Housing Affordability used in Housing Research: A Qualitative Study

Supplementary Data

S1 Table: Review Design

Criteria for Exclusion and Inclusion			Source of Data	Data Collection Technique	Databases
	Exclusion	Inclusion			
Timeline	Not Within 2000 – 2018	Within 2000 – June, 2018	Print (Hard); Online (Soft).	Title, Abstract, Keywords, Key arguments, Research methodologies, Conclusions and Findings.	Web of Science, Google Scholar, Springer, ProQuest Central.
Nature of Publication	Non-Peer Reviewed publications, Book Chapters, Book Reviews, Non-Empirical articles, Monographs, News items, Duplicates, Editorials and Encyclopedia articles.	Peer-Reviewed publications and Reports of Empirical Studies.			
Theme	Regression Models and Econometric Analysis Approaches, as well as Indexes formulated by professional bodies.	Normative methods, Basic Measurements Approaches and Concepts of Housing Affordability, as well as Mathematical Models (basically adaptations from the basic approaches with more robust methodologies).			
Language	Non-English Publications	English or Translated in English			
Search Scope	Multidisciplinary and Wide-ranging				
Search method	Verbatim, Boolean and Word Combination				
