# Supplementary material for: Methodological weaknesses in the measurement approaches and concept of housing affordability used in housing research: A qualitative study
Source: PLoS One. 2019 Aug 30;14(8):e0221246. doi: 10.1371/journal.pone.0221246 (PMC6716639; doi:10.1371/journal.pone.0221246)
Supplement: S2 Table — (PDF) [file pone.0221246.s002.pdf]

Methodological Weaknesses in the Measurement Approaches and Concept of Housing Affordability used in Housing Research: A Qualitative Study

Supplementary Data

S2 Table: Extraction sheet for source type and database sources included in the study.

S/N	Definition, Meaning and Concept	Measures, approaches and Indicators/Standards/Trends and Challenges	Other Relevant and Housing-Related materials	Title	Year	Context	Methodology	Source Type	Database	Major Finding
1	Gabriel S. Painter G.			Why affordability matters	2018	USA	Quantitative	Journal Article (Regional Science and Urban Economics)	Web of Science	The study identified the several channels by which family or individual outcomes become inferior when rents increases are not in accordance with that of income. Findings suggest that growing rent burdens could impede economic prospects of cities.
2	Taltavull P, Juárez F.			Housing affordability. A literature review	2012	Spain	Qualitative	Journal Article (Galega Magazine of Economics)	Web of Science	The research estimated a few ratios of affordability for Spain and discussed some relationship with business cycle.
3		Jewkes M, Delgadillo L M.		Weaknesses of housing affordability indices used by practitioners.	2010	USA	Qualitative	Journal Article (Journal of Financial Counseling and Planning)	Web of Science	The study reviewed the three most common applied indices of housing affordability in the United States. It revealed the limited ability in the adaption of measures of housing affordability to individual households.
4		Arman M, Zuo J, Wilson, L, Zillante G, Pullen S.		Challenges of responding to sustainability with implications for affordable housing.	2009	Australia	Qualitative	Journal Article	Google Scholar	Using the application of sustainability to affordable housing in Australia as a case study, the study argues that instead of limiting sustainable outcomes, the continuation of the sustainability debate which centers on several challenges is an indication that sustainability could be attained.
5		Dong H.		The impact of income inequality on rental affordability: An empirical study in large American metropolitan areas.	2018	America	Quantitative	Journal Article (Urban Studies)	Web of Science	The study evaluated the effect of income inequality on low-income tenant households' rental affordability in 100 largest metropolitan areas of America. The cross-sectional analyses reveal that, a 0.1 increase in Gini coefficient was associated with 2.2 and 4.4 percentage points of more low-income with worst case

										rent-burdened households in 2000 and 2008–2012, respectively.
6		Mulliner E, Smallbone K., Maliene V.		An assessment of sustainable housing affordability using a multiple criteria decision making method	2013	UK	Empirical	Journal Article (Omega)	Web of Science	The study presented the application of a novel methodology of housing affordability assessment. The developed methodology considers a range of environmental, economic and social criteria. .
7		Mulliner E, Malys N, Maliene V.		Comparative analysis of MCDM methods for the assessment of sustainable housing affordability	2016	UK	Empirical	Journal Article (Omega)	Web of Science	The study illustrated the application of WSM, WPM. TOPSIS, revised AHP, COPRAS models of the MCDM for sustainable housing affordability assessment. It analyzed 20 evaluative criteria used and performed a comparison of several MCDM methods.
8		Cheong T S, Li J.		Transitional distribution dynamics of housing affordability in Australia, Canada and USA	2017	USA, Canada and Australia,	Empirical	Journal Article (International Journal of Housing Markets and Analysis)	Web of Science	The study explored indicators of transitional dynamics of housing affordability in major metropolis of 3 advanced countries; adopting an innovative approach.
9		Wegmann Jake		Measuring What Matters: A Call for a Meaningful Metric of Affordable Rental Housing Production Cost-Efficiency.	2014	USA	Quantitative	Journal Article (Housing Policy Debate)	Web of Science	Proposed a replacement metric, the subsidy per housing affordability equivalent (SHARE) ratio.
10		Luckey Kara S.		Affordable for whom? Introducing an improved measure for assessing impacts of transportation decisions on housing affordability for households with limited means.	2018	USA	Quantitative	Journal Article (Research in Transportation Business & Management)	Web of Science	Introduced the location-sensitive residual income (LSRI) approach – which reflects the realities facing households more fully by incorporating differences associated with household income, composition, childcare requirements, and residential location.
11		Stone ME.		What is housing affordability? The case for the residual income approach.	2006a	USA and UK	Qualitative	Journal Article (Housing policy debate)	Web of Science	The paper highlighted some potential implications of putting into consideration the residual income paradigm for housing problems and needs analysis, for mortgage underwriting practice and for housing subsidy policy.
12		Nwuba CC,		Measuring housing	2018	Nigeria	Qualitative	Journal Article	Google	Presented descriptions of normative measures

		Kalu IU.		affordability: the two approaches.				(ATBU Journal of Environmental Technology)	Scholar	from perspectives of different authors and the ongoing debate on their relative suitability as affordability measures.
13		Mulliner E, & Maliene V.		An analysis of professional perceptions of criteria contributing to sustainable housing affordability	2014	UK	Qualitative	Journal Article (Sustainability)	Web of Science	The study developed, analyzed and validated an all-inclusive system of criteria representing sustainable housing affordability.
14		Mattingly K, Morrissey J.		Housing and transport expenditure: socio-spatial indicators of affordability in Auckland	2014	USA	Quantitative	Journal Article (Cities)	Web of Science	Findings show that methods of measuring housing affordability tend to disregard cost of transportation. Incorporating cost of commuting<br>Generated considerable different affordability pattern. Houses in the outskirts of town were revealed to be somewhat less expensive.
15		Aribigbola A.		Housing affordability as a factor in the creation of sustainable environment in developing world: the example of Akure, Nigeria.	2011	Nigeria	Quantitative	Journal Article (Journal of Human Ecology)	Web of Science	Housing affordability is considered essential in the creation of sustainable environment.<br>Findings reveal that affordability is one of the major problem associated with urban housing.
16	Makinde O.			Housing delivery system, need and demand	2014	Nigeria	Qualitative	Journal Article (Environment, Development and Sustainability)	Web of Science	Reviewd the past and current housing delivery programmes in Nigeria and analysed the demand and supply issues; as well as factors responsible for high housing pricing trends.
17	Howenstine EJ.			Attacking housing costs: foreign policies and strategies.	1983	USA	Qualitative	Book	Google Scholar	The book sets out the affordability problem by comparing between countries the trends in building costs, with a focus on labor and material costs, as well as rents. It also examined the methods applied in a number of countries in checking the rising costs problems; policies designed for increased production, financial cost reduction and making it more available to purchasers and suppliers, and reducing the cost and increasing the availability of land.
18	Maclennan D, Williams R.			Affordable housing in Britain and America	1990	UK and USA	Quantitative	Book	Google	The book suggested an affordability definition and made a comparative of discussions on affordable housing in two countries
19	Bramley G.			An affordability crisis in British housing: dimensions, causes	1994	Britain	Qualitative	Journal Article (Housing Studies)	Web of Science	Housing system in Britain suffered affordability crisis in the late 1980s and early 1990s, appeared in many dissimilar ways, and comparable to those experienced in other

				and policy impact.						countries.
20	Whitehead CM.			From need to affordability: an analysis of UK housing objectives.	1991	UK	Qualitative	Journal Article (Urban Studies)	Web of Science	The paper discusses the principles that lie behind the affordability and need concepts and the manner they have been defined.
21	Hancock KE.			Can Pay? Won't Pay?'or Economic Principles of'Affordability'	1993	UK	Quantitative	Journal Article (Urban Studies)	Web of Science	The paper considers what meanings have been given to the term affordability in practice and suggests a range of analytically more useful definitions. It argues from economic first principles that it is more logical to use some form of residual income definition than one based on a prescribed ratio of housing costs to income.
22		Thalmann P.		‘House poor’ or simply ‘poor’?.	2003	Switzerland	Empirical	Journal Article (Journal of Housing Economics)	Web of Science	The study defined a set of indicators for distinguishing problems of housing affordability resulting from low income from those chiefly arising from high rents. In place of the common rent to income ratio, it used a residual income indicator with over-consumption and over-paying indicators for housing services.
23		Burke T, Ralston L.		Measuring housing affordability.	2004	Australia	Qualitative	Report/ Document	Google Scholar	Conventional fixed cordon measures could wrongly estimate the scale of housing stress. Results show that between 1975 and 1999 in the lowest two income quintiles; housing affordability reduced for low income private and public renters based on the conventional 25/30 per cent income ratio measure.
24		Leishman C, Rowley S.		Affordable housing	2012	UK	Qualitative	Book	Google Scholar	The study reviewed the housing affordability causes and definitions as policy relevant to housing market and social issues.
25	Minchenko MM, Nozdrina NN.			The Dynamics of Housing Affordability for the Population of Russia in 2008–2014	2017	Russia	Quantitative	Journal Article (Studies on Russian Economic Development)	Web of Science	The paper discussed the affordability dynamics of the Russia’s population of home ownership purchase between the two crises of 2008 and 2014. Findings show that in Russia after the crisis of 2008 the financial affordability of housing increased in all indices considered.
26		Chapman P.		Housing affordability in Australia	2006	Australia	Qualitative	Report/ Document	Google Scholar	The article posited that a number of measures of housing affordability are in use. Whichever is used, significant numbers of Australian households are found ro have housing affordability problems and these are persisting over time
27	Rowley S, Ong R.			Housing Affordability, Housing Stress and Household Wellbeing in Australia.	2012	Australia	Quantitative	Report/Document	Google Scholar	The report evaluated a possible link between housing stress measures, using the 30:40 rule and other social and economic measures of well-being.

28	Quigley JM, Raphael S.			Why isn't it more affordable?.	2004	USA	Quantitative	Journal Article (Journal of Economic Perspectives)	Web of Science	This paper reviews trends in housing affordability in the US over the past four decades.
29		Stone ME.		Shelter Poverty: New Ideas on Housing Affordability	1993	USA	Quantitative	Book	Google Scholar	The research developed a measure termed shelter poverty, with which can demonstrate quantitatively the gap between the cost of housing and income levels.
30	Ken Maher			Housing affordability: Why architecture matters.	2017	Australia	Qualitative	Report/ Document	Google Scholar	The report discussed housing affordability issues with over 50 Australian parliament members. It highlighted the important role architecture profession plays in delivering affordable living and in the overall development of the built environment.
31		Napoli G.		Housing Affordability in Metropolitan Areas. The Application of a Combination of the Ratio Income and Residual Income Approaches to Two Case Studies in Sicily, Italy.	2017	Italy	Empirical	Journal Article (Buildings)	Web of Science	The paper proposed a methodology for assessing threshold-income as an index for housing affordability measurement by combining the ratio income and residual income approaches.
32		Stone M, Burke T, Ralston L.		The residual income approach to housing affordability: the theory and the practice	2011	Australia	Quantitative	Report/Document	Google Scholar	The report suggested the works required at level of methodological detail not conducted in comparable studies in Australia or internationally
33		Fisher LM, Pollakowski HO, & Zabel J.		Amenity-Based Housing Affordability Indexes	2009	USA	Quantitative	Journal Article (Real Estate Economics)	Web of Science	The study developed a unique measure of area affordability that describes the distinctive nature of housing supply that is deemed affordable in different locations of a city to different households.
34		Isalou AA., Litman T, Shahmoradi B.		Testing the housing and transportation affordability index in a developing world context: A sustainability comparison of central and suburban districts in Qom, Iran.	2014	Iran	Quantitative	Journal Article (Transport policy)	Web of Science	The study the overall transport and housing costs for several locations in Iran. It maintained that affordability must reflect housing and transport costs combined. Findings reveal that households in suburban locations spend much more than those at central locations.
35	Desmond M.			Heavy is the house: Rent burden among the American Urban Poor.	2018	U.S.A	Quantitative	Journal Article (International Journal of Urban and Regional	Web of Science	Argues that housing is essential in the lives of poor urban dwellers, yet is still marginal to the American inequality sociology.

								Research)		
36	HUD			WORST CASE HOUSING NEEDS 2011 REPORT TO CONGRESS	2011	USA	Qualitative	Report/Document	Google Scholar	Examined the causes of and trends in worst case needs for affordable rental housing.
37		Dong H.		The impact of income inequality on rental affordability: An empirical study in large American metropolitan areas.	2018	America	Quantitative	Journal Article (Urban Studies)	Web of Science	The study evaluated the effect of income inequality on low-income tenant households' rental affordability in 100 largest metropolitan areas of America. The cross-sectional analyses reveal that, a 0.1 increase in Gini coefficient was associated with 2.2 and 4.4 percentage points of more low-income with worst case rent-burdened households in 2000 and 2008–2012, respectively.
38	Cox W.			What Is Middle-Income Housing Affordability	2018a	Australia	Qualitative	Report/ Document	Google Scholar	The report gave a perspective on housing affordability including ratings and data for almost 300 cities in nine countries.
39	Dewilde C.			Explaining the declined affordability of housing for low-income private renters across Western Europe.	2018	Western Europe	Quantitative	Journal Article (Urban Studies)	Web of Science	The study explained the housing affordability trends in private rented sector for lower-income households across Western European nations, from a demand versus supply perspective. Findings reveal that across Europe affordability declined as a result of demand pressure caused by in-migration.
40		Li VJ, Cheng AWW, & Cheong TS.		Home purchase restriction and housing price: A distribution dynamics analysis	2017	China	Empirical	Journal Article (Regional Science and Urban Economics)	Web of Science	The paper employed distribution dynamics analysis and mobility probability plot. It found that in curbing speculative investment demand that home purchase restriction has considerable impact; and it is most efficient for housing with 5% monthly price increase. And concluded that removing home purchase restriction may increase levels of housing price.
41	Yang H.			An Analysis of Restrictions on Housing Purchases in China	2017	China	Qualitative	Report/Document	Google Scholar	The paper analyzed the reasons for high prices of housing in China. It concludes that the reasons are not supply and demand related, but rather problems of financial and fiscal.
42		Sarı ÖBÖ, Khurami EA.		Housing affordability trends and challenges in the Turkish case.	2018	Turkey	Quantitative	Journal Article (Journal of Housing and the Built Environment)	Web of Science	The study examined the housing affordability trends and challenges in the Turkey. The findings show that different approaches weakly agree in identifying the affordability problem, and a promising method for a thorough understanding of the issue is subjective approach.
43	Nwuba CC, Kalu IU, Umeh JA.			Determinants of homeownership affordability in Nigeria's urban housing markets	2015	Nigeria	Empirical	Journal Article (International Journal of Housing Markets and Analysis)	Web of Science	Findings show that savings, household income, education and construction period are homeownership determinants of affordability with positive impact. Conversely, cost of land, household size, current rental housing expenditures, building cost inflation, building

										cost relative to income and non-housing expenditures are affordability determinants with negative impact.
44	Nwosa PI.			Fiscal Policy and Exchange Rate Movement in Nigeria.	2017	Nigeria	Qualitative	Journal Article (Acta Universitatis Danubius: Oeconomica)	ProQuest Central	The study shows that variables of fiscal policy were statistically significant in influencing Nigeria’s exchange rates, using the Ordinary Least Square (OLS) and the regression estimate. The study suggested that variables of fiscal policy in Nigeria are significant determinants of exchange rate movement.
45		O’Dell W, Smith MT, White D.		Weaknesses in current measures of housing needs.	2004	USA	Qualitative	Journal Article (Housing and Society)	Web of Science	The study examined the current weaknesses in housing needs measurement and proposed modifications to the identified measures. Results suggest that problems of housing condition are more localized than problems of cost burden. Implying that measures are required at neighborhood level geographies.
46	Duffy, D.			A Note on Measuring the Affordability of Homeownership.	2004	Ireland	Qualitative	Report/ Document	Google Scholar	The study remarked that though The housing affordability seems to be a relatively simple concept it is not without some difficulties and that alternative measures of the affordability of homeownership can show a different picture for the same time period.
47			Eakes M.	Preserving the American dream: Predatory lending practices and home foreclosures.	2007	USA	Qualitative	Report/ Document	Google Scholar	The document argues that the issues of the US markets were made worse due to the low-quality credit that was provided in the early 2000s.
48	Abelson P.			Affordable housing: concepts and policies	2009	Australia	Quantitative	Journal Article (Economic papers: a journal of applied economics and policy)	Web of Science	Evaluates the housing affordability concept, policies and current measures for increasing housing affordability. The study defines costs of housing in nominal instead of real terms and incorporates mortgage repayments that are referred to as savings.
49		Abeysinghe T, Gu JY.		Lifetime Income and Housing Affordability in Singapore	2011	Singapor e	Quantitative	Journal Article (Urban Studies)	Web of Science	Discusses the most commonly used measures of housing affordability that compare current income with house prices or housing costs. It concludes that in spite of literature emphasis on the importance of long-term affordability, researchers are still unable to innovate measures of lifetime income due to limited data.
50	Rowley S, Ong R, Haffner M.			Bridging the gap between housing stress and financial stress: The case of Australia.	2015	Australia	Quantitative	Journal Article (Housing Studies)	Web of Science	The study showed that the application of longitudinal data can improve the housing stress measure and provides more accurate relationship assessment between housing costs and financial well-being.
51		Linneman		Housing	1992	USA	Quantitative	Journal Article	Web of	This paper focuses prithesis of

		PD, Megbolugbe IF		affordability: myth or reality?.				(Housing Studies)	Science	major trends/changes in the the-marily on housing affordability. The auoretical and empirical investigation of the thors rely primarily on the US experience housing market as well as with exploring to articulate the affordability issue, but the impacts of public policy interventions draw examples of public policy intervenon urban housing markets.
52		Gan Q, Hill RJ.		Measuring housing affordability: Looking beyond the median.	2009	Australia	Empirical	Journal Article (Journal of Housing economics)	Web of Science	Empirically discovered that the dissimilarity between repayment and purchase affordability can be significant. It suggested how median house-price-to-income ratio measures could be modified to consider the whole distribution of house prices and income
53		Kutty NK.		A new measure of housing affordability: Estimates and analytical results.	2005	USA	Quantitative	Journal Article (Housing policy debate)	Web of Science	The paper introduced the housing-induced poverty concept, which is similar to the concept of shelter poverty developed by Stone 1993.
54		Yang Z, Shen Y.		The affordability of owner occupied housing in Beijing.	2008	China	Qualitative	Journal Article (Journal of Housing and the Built Environment)	Web of Science	The study defined a new measure of affordability by residual income.
55		Duan M.		Investigation on housing affordability in Lanzhou, Northwest China	2011	China	Quantitative	Journal Article (International Journal of Housing Markets and Analysis)	Web of Science	This paper aims to provide an insight into the housing affordability by analyzing the influence factors of purchasing standard housing in Lanzhou, which is the second largest city in Northwest China.
56		Yang Z, Yi C, Zhang W. Zhang C.		Affordability of housing and accessibility of public services: evaluation of housing programs in Beijing	2014	China	Quantitative	Journal Article (Journal of Housing and the Built Environment)	Web of Science	The study analyzed the correlation of affordable housing program with household housing affordability and public services accessibility, in Beijing. It studied Economic and Comfortable Housing based on eligible household unique database and affordable housing projects. Findings show that the program does not make housing accessible and affordable for eligible households.
57		Revington N, Townsend C.		Market rental housing affordability and rapid transit catchments: Application of a new measure in Canada.	2016	Canada	Empirical	Journal Article (Housing policy debate)	Web of Science	This study operationalizes a residual income approach to identify market rental housing that is affordable to two household configurations (couples with children and couples without children) in two categories below the median income.
58		Mundt, A.		Housing benefits and minimum	2018	Australia	Empirical	Journal Article (International	Web of Science	Findings indicate that in Vienna and environs, and some other major Austrian cities, overall

				income schemes in Austria–an application of the residual income approach to housing affordability of welfare recipients.				Journal of Housing Policy)		benefits fail to cover common costs of housing, leading to insufficiency of funds necessary for non-housing expenditure.
59		Yip NM.		"Housing affordability in England".	1995	UK	Empirical	Dissertation	Google Scholar	Findings suggest that, about a quarter of households in 1991 were in unaffordable housing when assessed with the ratio measurement and the traditional residual income measurement.
60		Henman P, Jones A.		Exploring the use of residual measures of housing affordability in Australia: Methodologies and concepts	2012	Australia	Qualitative	Report/ Document	Google Scholar	The report explored the advantages of more application of residual measures of housing affordability as an alternative of the ratio measures as well as the possibilities for more housing consumption outcome measures.
61		Ndubueze O.		Measuring housing affordability: A composite approach.	2007	Nigeria	Empirical	Conference Paper	Google Scholar	Developed a composite affordability index that combined and modified the housing expenditure-to-income ratio (adjusted with housing quality) and shelter-poverty affordability measure using relevant data from the Nigeria Living Standards Survey 2003-2004 (NLSS) database. Findings reveal that the aggregate approach addresses wider dimensions of housing affordability more than conventional ratio measures.
62	McCord M, McGreal S, Berry J, Haran M., Davis P.			The implications of mortgage finance on housing market affordability	2011	UK	Quantitative	Journal Article (International Journal of Housing Markets and Analysis)	Web of Science	Findings show that the mortgage finance-affordability relationship is controlled by the mortgage market deregulation contributing to the rise in affordability pressures and house prices during the market up cycle.
63		Haffner M, Heylen K.		User costs and housing expenses. Towards a more comprehensive approach to affordability	2011	Netherlands	Quantitative	Journal Article (Housing Studies)	Web of Science	Reviewed the different definitions and concept of affordability. Results show that each concepts of short-term and long-term affordability has its own uses and cannot be interchanged.
64		Taltavull P, Tang CP.		Measuring the affordability of housing association rents in England: a dual approach	2012	UK	Empirical	Journal Article (International Journal of Housing Markets and Analysis)	Web of Science	The analysis of rent-to-income ratio suggested that housing association rents were affordable in general. But, the analyses of residual income using two different minimum acceptable standards created some doubts.
65		Thalmann P.		Identifying households which need housing	1999	Switzerland	Empirical	Journal Article (Urban Studies)	Web of Science	Findings show that for every three households more than two that suffer high rent burdens can afford decent housing.

				assistance						
66		Ndubueze O.		Urban housing affordability and housing policy dilemmas in Nigeria	2009	Nigeria	Empirical	Dissertation	Google Scholar	An update of Ndubueze (2007).
67		Sunega P, Lux M.		Subjective perception versus objective indicators of overcrowding and housing affordability	2016	EU States	Quantitative	Journal Article (Journal of Housing and the Built Environment)	Web of Science	The findings indicate that alternative threshold definitions could reduce the current discrepancy between objective indicators and subjective perceptions of overcrowding and housing affordability.
68		Mulliner E K.		A model for the complex assessment of sustainable housing affordability	2012	UK	Empirical	Dissertation/ Thesis	ProQuest Dissertations and Theses Global	The research evaluated the housing affordability concept and assessment. It provided a new knowledge by applying and developing a complex model for sustainable housing affordability assessment.
69	Feins J, White JR, Charles S			The Ratio of Shelter Expenditure to Income: Definitional Issues, Typical Patterns and Historical Trends	1977	USA	Qualitative	Report/ Document	Google Scholar	Posited that the rule [of thumb] is both inaccurate and inappropriately use.
70	Maclennan D, Gibb K, More A.			Paying for Britain's housing	1990	UK	Quantitative	Book	Google	The book offered insights into the housing affordability trends and challenges in Britain.
71		Wood G, Ong R, Dockery AM.		The long-run decline in employment participation for Australian public housing tenants: an investigation.	2009	Australia	Empirical	Journal Article (Urban Studies)	Web of Science	This paper explores the potential causes of this trend and their policy implications.
72	Grinstein-Weiss M, AN Chowa G, Casalotti, AM.			Individual development accounts for housing policy: Analysis of individual and program characteristics	2010	USA	Quantitative	Journal Article (Housing Studies)	Web of Science	The paper examined the outcome of savings of participants of IDA who saved in the American Dream Policy Demonstration, for a home. Findings show that when provided structured opportunities, low income IDA participants can successfully save.
73		Olanrewaju A, Woon TC.		An exploration of determinants of affordable housing choice.	2017	Malaysia	Empirical	Journal Article (International Journal of Housing Markets and Analysis)	Web of Science	The paper shows that financial factor, general factor, building factor, accessibility factor, income factor, location factor and market factor are affordable housing choice determinants, which is useful to homebuyers, developers and policy makers towards delivery of affordable housing in Malaysia.
74		Kearns A,		Community	1996	UK	Quantitative	Report/	Google	The research analyzed the impact of a rent

		Gibb K, More A, Keoghan M, Pryce G.		ownership rents: Greater Easter-house in the Glasgow context.				Document	Scholar	increase on labor market incentives in the UK. Findings show the work-disincentive impacts of Housing Benefit, using a priori simulations of levels of threshold wage to show that increasing rents have strong disincentive impact on the will to work.
75		Chasco C, Gallo JL.		The impact of objective and subjective measures of air quality and noise on house prices: a multilevel approach for downtown Madrid	2013	Spain	Empirical	Journal Article (Economic Geography)	Web of Science	Evaluated the effect of Air quality and urban noise on the transaction prices in downtown Madrid. Employing both subjective and objective measures of noise and air quality as well as multilevel models. Findings reveal that air and pollution noise are variables of place-based perception with a supposed halo effect.
76			Stiglitz J, Sen AK, Fitoussi JP.	The measurement of economic performance and social progress revisited: reflections and overview	2009	France	Quantitative	Report/ Document	Google Scholar	The purpose of this Report is to take stock of what has already been done, to assess the weaknesses (and strengths) of current metrics, and to propose some modifications to the existing statistical apparatus.
77		OECD		"How's Life? at a Glance", in How's Life?: Measuring Well-being,	2013	OECD countries	Qualitative	Report/Document	Google Scholar	The study presented strengths and weaknesses diagnosis of countries well-being. The findings show that OECD countries have progresses remarkably in many areas of well-being in the last 20 years. However, there exists inequality in progress across the 11 dimensions as seen in the OECD well-being frame work.
78		Robinson M, Scobie GM, Hallinan B.		Affordability of housing: concepts, measurement and evidence	2006	New Zealand	Empirical	Journal Article (Month)	Web of Science	Results show that the difficulty in affordability definition and that there is no consensus on its measurement, and that using a range of measures, the study examined the trends over time. It revealed no long-term affordability trend when all measures are considered.
79			Mardani A, Jusoh A, MD Nor K, Khalifah Z, Zakwan N, Valipour A.	Multiple criteria decision-making techniques and their applications–a review of the literature from 2000 to 2014	2015	Malaysia	Qualitative	Journal Article (Economic Research)	Web of Science	The paper systematically reviewed the methodologies and applications of the MCDM approaches and techniques.
80		Said R, Majid RA, Alias A, Adnan YM., Razali M N.		Sustainable housing affordability in Sabah	2016	Malaysia	Empirical	Journal Article (Planning Malaysia Journal)	Web of Science	The results suggest that locations with high degree of utility are the best area that is consistent with factors of sustainable housing affordability. So also, locations with a lower ranking degree in utility can be referred to as a worst-performing location.
81			Zavadskas E K, Cavallaro,	MCDM assessment of a healthy and	2017	Lithuania	Quantitative	Journal Article (Sustainability	Web of Science	The paper in the search for a more reliable tool, proposed a system of MCDM methods to

			F, Podvezko V, Ubarte I, Kaklauskas A.	safe built environment according to sustainable development principles: A practical neighborhood approach in Vilnius.				)		be applied to a single problem, since alternative MCDM methods when applied to the same problem often produce different results.
82		Daniel L, Baker E, Lester L.		Measuring housing affordability stress: can deprivation capture risk made real?.	2018	Australia	Empirical	Journal Article (Urban Policy and Research.)	Web of SciencE	Explored material deprivation as a compelling approach to capturing the additional experience and individual impacts of housing affordability problems
83		Ben-Shahar D, Warszawski J.		Inequality in housing affordability: Measurement and estimation	2016	Isreal	Quantitative	Journal Article (Housing Studies)	Web of Science	This research proposes and examines a new measure for assessing the state of housing affordability inequality.
84			Wang XJ, Zhang JY, Shahid S, ElMahdi A, He RM, Wang XG, Ali M.	Gini coefficient to assess equity in domestic water supply in the Yellow River	2012	China	Quantitative	Journal Article (Mitigation and Adaptation Strategies for Global Change)	Web of Science	The paper developed a method to calculate the Gini coefficient of water use as an indicator to measure the equality in domestic supply of water. The findings reveal that in the Yellow River basin after 2001 there is a declining drift in Gini coefficient of domestic water use implying that in the basin the use of domestic water is becoming more equitable.
85			Cheong TS, Wu Y.	Convergence and transitional dynamics of China's industrial output: A county-level study using a new framework of distribution dynamics analysis.	2015	China	Empirical	Journal Article (China Economic Review)	Web of Science	The study provided an empirical evidence on industrial output transitional dynamics by developing a novel technique of distribution dynamics analysis called mobility probability plot (MPP).<br>The new technique can remedy many weaknesses in the traditional display tools employed in the distribution dynamics literature.
86		Chen J, Hao Q, Stephens M.		Assessing housing affordability in post-reform China: a case study of Shanghai.	2010	China	Empirical	Journal Article (Housing Studies)	Web of Science	The study improved the conventional indicators to capture other aspects of affordability for new market entrants. The evidence generated in applying several indicators of affordability and the introduction of a dynamic element could inform policy more appropriately than the conventional measures by suggesting the duration, extent and nature of policy responses necessary for different groups of income.
87		Gabriel M, Jacobs K, Arthurson K,		Conceptualizing and measuring the housing	2005	Australia	Qualitative and Quantitative	Report/ Document	Google Scholar	The report reviewed relevant literature dealing on issues of affordability and discusses some of the key methods of measuring housing

		Burke T, Yates J.		affordability problem.						affordability.
88		Bramley G.		Affordability, poverty and housing need: triangulating measures and standards	2012	U.K	Quantitative	Journal Article (Journal of Housing and the Built Environment)	ProQuest Central	Appraised possible normative measures--subjective indicator relationship. The derived composite measure adopted the normative ratios and self-reported affordability dilemmas, and used subjective approach to validate the normative measures.
89	Talen E.			Affordability in new urbanist development: Principle, practice, and strategy.	2010	USA	Qualitative	Journal Article (Journal of Urban Affairs)	Web of Science	The findings indicate that while approaches based on design to diversity in New Urbanism are contained in the sphere of possibilities, the availability of developers eager to undertake a multi-pronged strategy composed of creative financing, good location, government self-determination, support, and innovation, is necessarily limited.
90		Ruming K, Gurran N, Randolph B.		Housing affordability and development contributions: New perspectives from industry and local government in New South Wales, Victoria and Queensland.	2011	Australia	Empirical	Journal Article (Urban Policy and Research)	Web of Science	The study explored the unique experiences and perspectives of a 18 developers sample in relation to 26 projects across Victoria, New South Wales and Queensland.
91	Lee, Chyi Lin, Reed Richard G.			The relationship between housing market intervention for first-time buyers and house price volatility	2014	Australia	Empirical	Journal Article (Housing studies)	Web of Science	Examined the relationship between housing market intervention based on first-time owner subsidies in a global city and the level of house price volatility in the broader market.
